# Multiple independent acquisitions of ACE2 usage in MERS-related coronaviruses

**DOI:** 10.1016/j.cell.2024.12.031

**Published:** 2025-02-07

**Authors:** Cheng-Bao Ma, Chen Liu, Young-Jun Park, Jingjing Tang, Jing Chen, Qing Xiong, Jimin Lee, Cameron Stewart, Daniel Asarnow, Jack Brown, M. Alejandra Tortorici, Xiao Yang, Ye-Hui Sun, Yuan-Mei Chen, Xiao Yu, Jun-Yu Si, Peng Liu, Fei Tong, Mei-Ling Huang, Jing Li, Zheng-Li Shi, Zengqin Deng, David Veesler, Huan Yan

**Affiliations:** 1 State Key Laboratory of Virology and Biosafety, College of Life Sciences, TaiKang Center for Life and Medical Sciences, Wuhan University; Wuhan, Hubei, 430072, China.; 2 Department of Biochemistry, University of Washington; Seattle, WA 98195, USA.; 3 Howard Hughes Medical Institute, University of Washington; Seattle, WA 98195, USA.; 4 State Key Laboratory of Virology and Biosafety, Wuhan Institute of Virology, Chinese Academy of Sciences; Wuhan, 430071, China.; 5 Guangzhou National Laboratory, Guangzhou International Bio Island; Guangzhou, 510005, China; 6 Hubei Jiangxia Laboratory; Wuhan, 430207, China.; 7 These authors contributed equally to this work.; 8 Lead contact

## Abstract

The angiotensin-converting enzyme 2 (ACE2) receptor is shared by various coronaviruses with distinct receptor-binding domain (RBD) architectures, yet our understanding of these convergent acquisition events remains elusive. Here, we report that two bat MERS-related coronaviruses (MERSr-CoVs) infecting *Pipistrellus nathusii* (P.nat), MOW15–22 and PnNL2018B, use ACE2 as their receptor, with narrow ortholog specificity. Cryo-electron microscopy structures of the MOW15–22/PnNL2018B RBD-ACE2 complexes unveil an unexpected and entirely distinct binding mode, mapping >45Å away from that of any other known ACE2-using coronaviruses. Functional profiling of ACE2 orthologs from 105 mammalian species led to the identification of host tropism determinants, including an ACE2 N432-glycosylation restricting viral recognition, and the design of a soluble P.nat ACE2 mutant with potent viral neutralizing activity. Our findings reveal convergent acquisition of ACE2 usage for merbecoviruses found in European bats, underscoring the extraordinary diversity of ACE2 recognition modes among coronaviruses and the promiscuity of this receptor.

## Graphical Abstract

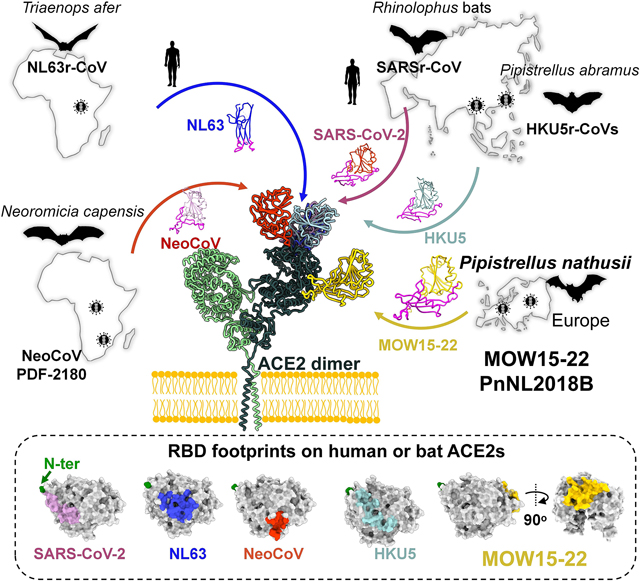

## INTRODUCTION

Human Middle East respiratory syndrome coronavirus (MERS-CoV) belongs to the *Merbecovirus* subgenus (also known as lineage C β-coronavirus or group 2c coronavirus) and is the causative agent of Middle-East respiratory syndrome (MERS) with a case-fatality rate of 36%.^1^ The evolutionary trajectory and host-switching history of MERS-CoV remain unclear. While dromedary camels are established intermediate hosts of MERS-CoV, increasing evidence from newly identified viral sequences indicates that Old World vesper bats (Vespertilionidae) are natural merbecovirus reservoirs.^2–7^ However, no currently known bat MERS-related coronaviruses (MERSr-CoVs) exhibit close similarity to human or camel MERS-CoV at the whole-genome level.^8,9^ NeoCoV, identified in African Laephotis bats (Cape serotine), represents the closest known relative to MERS-CoV, sharing 85.5% whole genome nucleotide sequence identity.^10^

Receptor usage determines host tropism and transmission of coronaviruses^11^. Dipeptidyl peptidase-4 (DPP4) has been documented as the entry receptor for several merbecoviruses, including MERS-CoV, HKU4, MjHKU4r, HKU25, and BtCoV-422.^12–18^ NeoCoV and the closely related PDF-2180 are two bat MERSr-CoVs that harbor markedly different spike (S) glycoproteins relative to that of MERS-CoV, and their cognate receptor remained elusive for a decade. For a long time, ACE2 was solely considered the host receptor recognized by members of sarbecoviruses (e.g., SARS-CoV-1 and SARS-CoV-2) and setracoviruses (e.g., NL63) through two different ACE2-binding modes with largely overlapping footprints in spite of their distinct receptor-binding domain (RBD) architectures.^19–23^ We recently revealed that NeoCoV and PDF-2180 also use the ACE2 receptor with a broad tropism across mammals but low human ACE2 (hACE2) usage efficiency.^9,24^ Cryo-electron microscopy (cryo-EM) analysis of the NeoCoV and the PDF-2180 RBDs bound to *Pipistrellus pipistrellus* (P.pip) ACE2 unveiled a distinct binding mode from that of SARS-CoV-1/SARS-CoV-2 and of NL63 involving extensive interactions with ACE2 N linked-glycans. The footprint of their RBDs shared few residues with that of SARS-CoV-1/2 or NL63, highlighting a convergent evolutionary history of ACE2 utilization in these viruses.^9^ This discovery suggested that a receptor switch might have occurred during MERS-CoV emergence in animals, such as bats and camels, potentially through recombination between a NeoCoV-like MERSr-CoV and a DPP4-using merbecovirus (e.g., HKU4 or BtCoV-422).^8,13^ Furthermore, these findings underscored the complexity of receptor binding modes used by merbecoviruses with diverse RBD sequences, many of which remain enigmatic.

Here, we discovered that two MERSr-CoVs circulating in European bats, designated MOW15–22 and PnNL2018B, utilize a subset of mammalian ACE2 orthologs as entry receptors. Cryo-EM analyses reveal that these viruses recognize ACE2 through an unprecedented binding mode, mapping >45Å away from the footprint of any other coronaviruses, emphasizing multiple independent acquisitions of ACE2 usage in bat MERSr-CoVs. This study sheds light on the global diversity and distribution of ACE2-using merbecoviruses and unexpected convergent evolution events, underscoring the zoonotic potential associated with these viruses.

## RESULTS

### Prediction of a distinct receptor recognition mode utilized by two MERSr-CoVs

**Figure 1.**
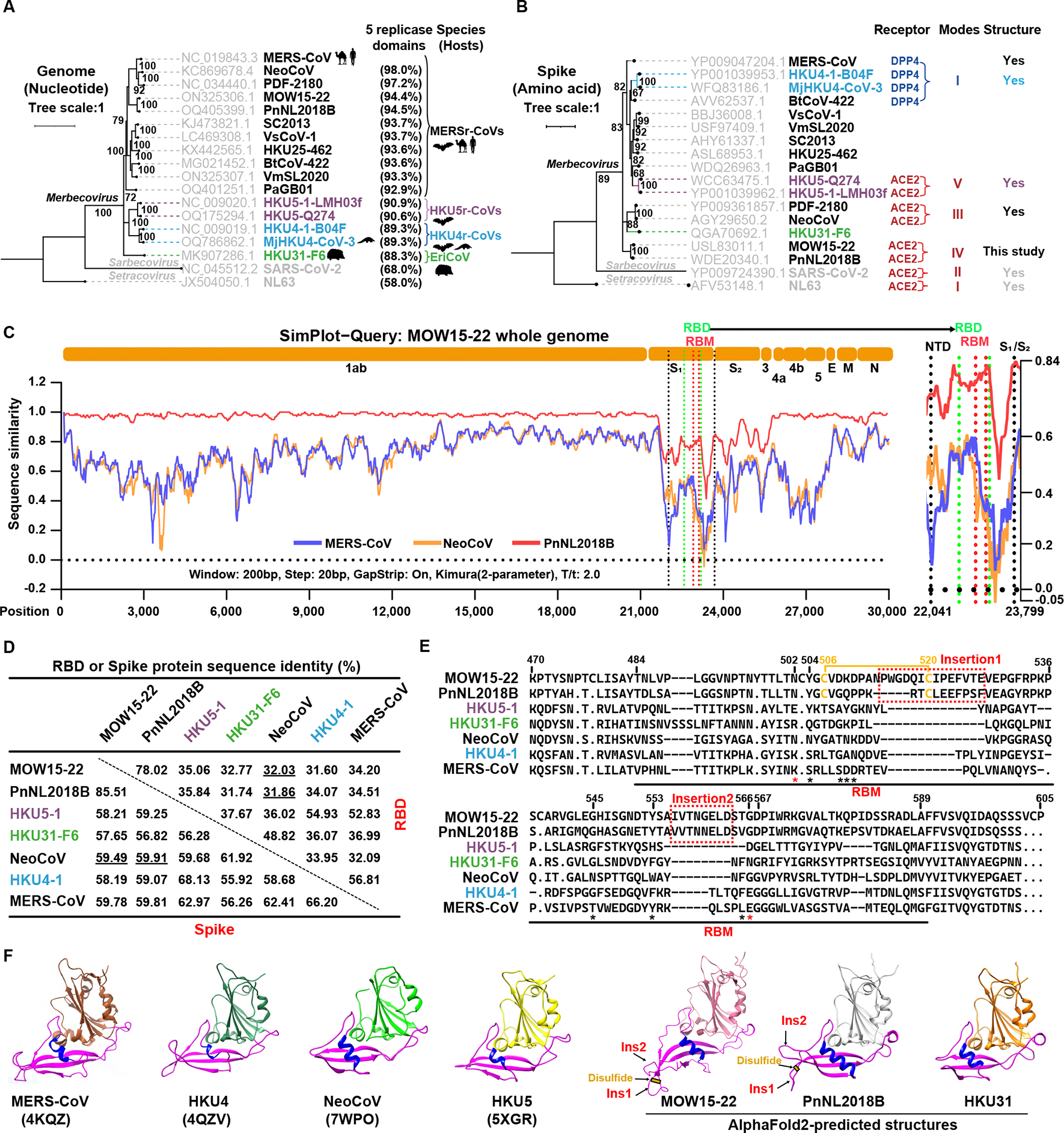
Prediction of a distinct receptor recognition mode utilized by two MERSr-CoVs. (**A-B**) Phylogenetic trees of representative merbecoviruses were generated using complete genome nucleotide sequences (A) or S glycoprotein amino acid sequences (B) with the IQ-tree method. NL63 was set as an outgroup. Amino acid sequence identities of five replicase domains (3CLpro, NiRAN, RdRp, ZBD, and HEL1) for coronavirus classification, host species, and receptor-related information (receptor usage, binding mode, and availability of RBD/receptor complex structure) are indicated. The scale bar represents 1 substitution per nucleotide/amino acid position. (**C**) Simplot analysis of the complete genome sequence similarity of several MERSr-CoVs analyzed based on the MOW15–22 genome. The right panel magnifies the RBD and adjacent regions. The boundaries of RBD (green) and RBM (red) coding regions are indicated by dotted lines. (**D**) Pairwise RBD and S amino acid sequence identities of indicated merbecoviruses. (**E**) RBM sequence alignment of the indicated merbecoviruses. MOW15–22 and PnNL2018B-specific insertions and disulfide are indicated in red and yellow, respectively. Red/black asterisks: residues crucial for NeoCoV interactions with P.pip ACE2 that are conserved/not conserved with MOW15–22 and PnNL2018B. The MOW15–22 residue numbering is shown. (**F**) Ribbon diagrams of experimentally determined structures or AlphaFold2-predicted structures of representative merbecovirus RBDs. Magenta indicates putative RBMs, and blue represents the RBM helix. Black arrows indicate the MOW15–22 and PnNL2018B-specific RBM insertions (Ins1 and Ins2). The yellow sticks represent the MOW15–22/PnNL2018B-specific disulfide bond in Ins1.

In light of the unexpected ACE2 usage of NeoCoV and PDF-2180, we investigated whether other merbecoviruses utilize ACE2 as a receptor. We conducted phylogenetic analyses of representative merbecovirus sequences, with a special emphasis on viruses belonging to MERSr-CoVs, as defined by amino acid sequence identity of the five concatenated replicase domains (3CLpro, NiRAN, RdRp, ZBD, and HEL1) greater than 92.4% compared with MERS-CoV (Figure 1A).^25^ At the whole-genome level, MOW15–22 and PnNL2018B (formerly PN-βCoV) are two bat MERSr-CoVs with the highest genetic similarity to MERS-CoV after NeoCoV and PDF-2180 (Figure 1A). At the S glycoprotein level, MOW15–22 and PnNL2018B cluster together and are distantly related to all other MERSr-CoVs. Hedgehog merbecovirus (Erinaceus coronavirus, EriCoV) HKU31 S shares high sequence identity with NeoCoV and PDF-2180, although HKU31 is not known to use ACE2 (Figure 1B).^26^ Simplot analysis comparing several viral genome sequences with MOW15–22 reveals that both MERS-CoV and NeoCoV are extensively divergent within the S_1_ subunit region (Figure 1C). Consistent with the phylogenetic tree based on S glycoproteins, pairwise analysis shows MOW15–22 and PnNL2018B share only 31–36% and 57–60% amino acid sequence identities with RBDs and S glycoproteins of other merbecoviruses, respectively (Figure 1D). Alignment of the region corresponding to the NeoCoV receptor-binding motif (RBM) reveals the presence of two insertions and a disulfide bond specific to MOW15–22 and PnNL2018B (Figure 1E). Only two (positions N502_MOW15–22_ and G566_MOW15–22_) out of nine NeoCoV residues critical for interactions with P.pip ACE2 are conserved with MOW15–22 and PnNL2018B (Figure 1E).^9^ Furthermore, the two insertions and elongated α-helix in the RBM present in AlphaFold2-predicted structures^26^ potentially affect receptor recognition (Figure 1F). Overall, these distinct sequence and structural features indicate that MOW15–22 and PnNL2018B may utilize a previously uncharacterized receptor and binding mode.

### MOW15–22 and PnNL2018B use ACE2 as receptor

**Figure 2.**
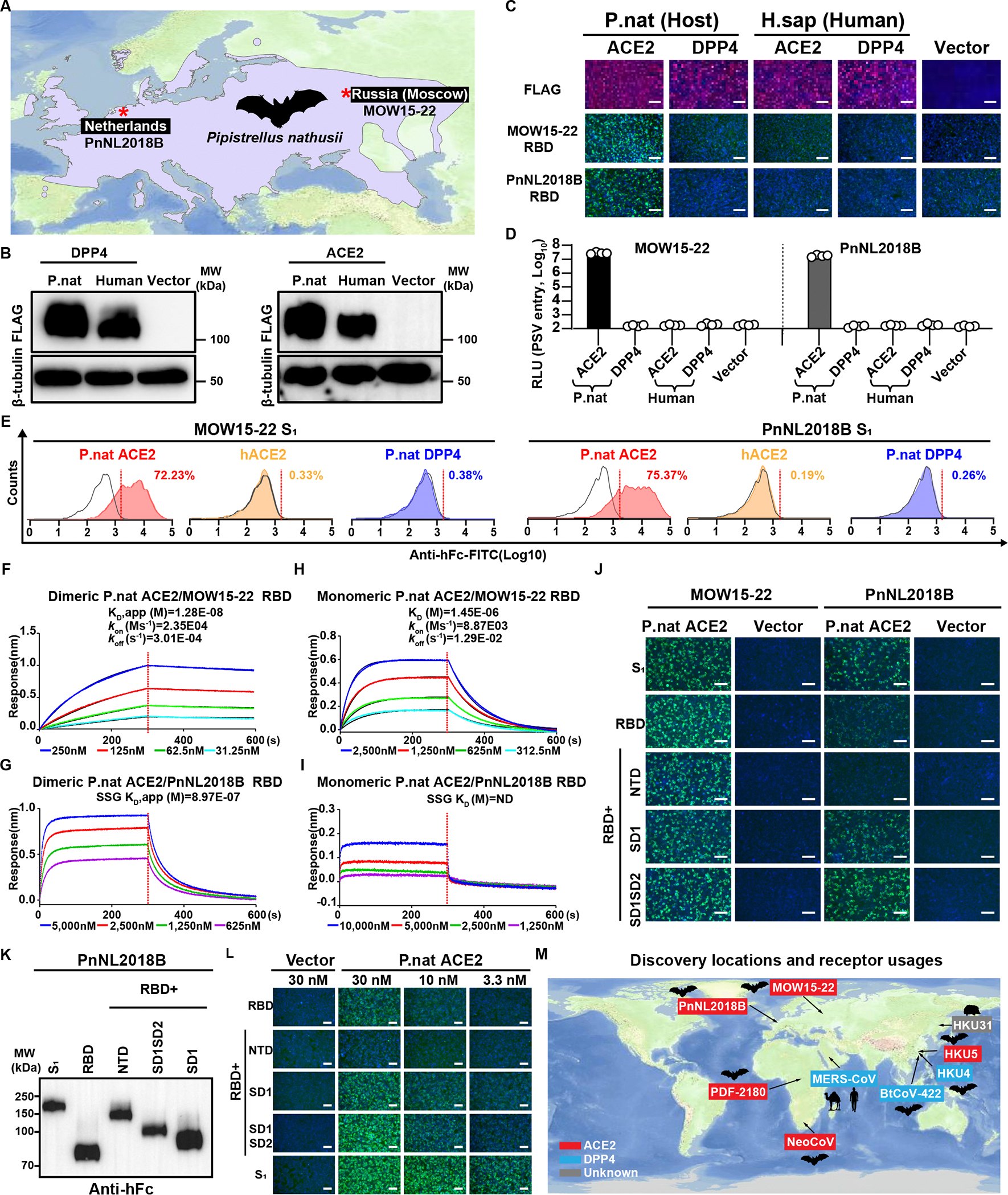
MOW15–22 and PnNL2018B use ACE2 as receptor. (**A**) Geographical distribution of *Pipistrellus nathusii* habitat (purple) in Europe. Data were retrieved from the IUCN (International Union for Conservation of Nature) Red List of Threatened Species, and the distribution chart was generated using Geoscene Pro. Red asterisks: discovery locations. (**B**) Expression levels of membrane-anchored human and P.nat ACE2 and DPP4 orthologs transiently transfected in HEK293T cells. (**C-D**) P.nat ACE2 but not P.nat DPP4 supports MOW15–22 and PnNL2018B RBD-hFc binding (C) and pseudovirus (PSV) entry (D) in HEK293T cells. Data are represented as mean ± SD for n=4 biological replicates. RLU: Relative light unit. (**E**) Flow cytometry analysis of MOW15–22 and PnNL2018B S_1_ binding to P.nat ACE2, hACE2 or P.nat DPP4 transiently expressed at the surface of HEK293T cells. White: vector control. Data shown are the mean of three technical repeats. (**F-I**) BLI analyses of binding kinetics of the dimeric (F-G) or monomeric (H-I) P.nat ACE2 ectodomains to immobilized monomeric biotinylated MOW15–22 RBD (F, H) or PnNL2018B RBD (G, I) immobilized on SA biosensors. Analysis was conducted with curve-fitting kinetic with global fitting (1:1 binding model) for F, H, and steady-state fitting for G. The fits are shown as black lines for F and H. ND: not determined. The color keys indicate the concentration of ACE2 used. (**J**) Binding of hFc-fused recombinant MOW15–22 and PnNL2018B RBDs and S_1_ subunits (4 μg/ml) to P.nat ACE2 transiently expressed at the surface of HEK293T cells and detected by immunofluorescence. (**K**) Expression levels of hFc-fused recombinant proteins comprising different domains of the PnNL2018B S_1_ subunit. Equal volumes of protein-containing supernatants used for purification were loaded for Western blot analysis. (**L**) Binding of the PnNL2018B RBDs and S_1_ subunits at various concentrations to HEK293T cells transiently expressing P.nat ACE2 analyzed by immunofluorescence. (**M**) Confirmed DPP4 or ACE2 usage of representative merbecoviruses is displayed with blue and red backgrounds, respectively. Discovery locations and natural hosts are indicated. Scale bars: 100 μm for C, J, and L.

MOW15–22 and PnNL2018B were recently sampled in *Pipistrellus nathusii* (P.nat, the common host) in Russia (Moscow region) and the Netherlands, respectively (Figure 2A).^27,28^ P.nat inhabits a wide range of Europe and undertakes seasonal long-distance migrations, typically from northeast to southwest Europe.^29–31^ Previous reports have proposed that DPP4 may serve as the entry receptor for MOW15–22 and PnNL2018B based on molecular docking analyses.^27,28^

Given the unique sequence and structural features of MOW15–22 and PnNL2018B, we set out to investigate their receptor usage by assessing the ability of human and P.nat ACE2 and DPP4 orthologs to support S-mediated pseudovirus entry and viral antigen binding. We found that P.nat ACE2, but not human ACE2 (hACE2), human DPP4, or P.nat DPP4, promoted MOW15–22 and PnNL2018B pseudovirus entry and RBD or S_1_ binding (Figures 2B–2E). Biolayer interferometry (BLI) analysis showed that the soluble dimeric P.nat ectodomain bound to the immobilized MOW15–22 and PnNL2018B RBDs with apparent affinities (K_D_, app) of 12.8 nM and 897 nM, respectively (Figures 2F and 2G). Furthermore, the monomeric P.nat ACE2 ectodomain bound to the MOW15–22 RBD with a low affinity of (K_D_) of 1.45 μM whereas we could hardly detect binding to the PnNL2018B RBD (Figure 2H and 2I). Investigation of the contributions of the four domains present in the S_1_ subunit revealed that the MOW15–22 RBD is sufficient for binding to P.nat ACE2 whereas the SD1 and SD2 domains (also named C and D domains^32^), but not the NTD (A domain), seem required for better PnNL2018B engagement (Figures 2J–2L). Collectively, these data along with our companion manuscript and another report showing that HKU5 uses *Pipistrellus abramus* (P.abr, the host) ACE2 and a few other ACE2 orthologs as entry receptors,^33,34^ expand the geographic distribution of ACE2-using merbecoviruses to all three continents of the Old World, with Pipistrellus bats as important reservoir hosts that should be closely monitored (Figure 2M).

### Multi-species ACE2 tropism of MOW15–22 and PnNL2018B

**Figure 3.**
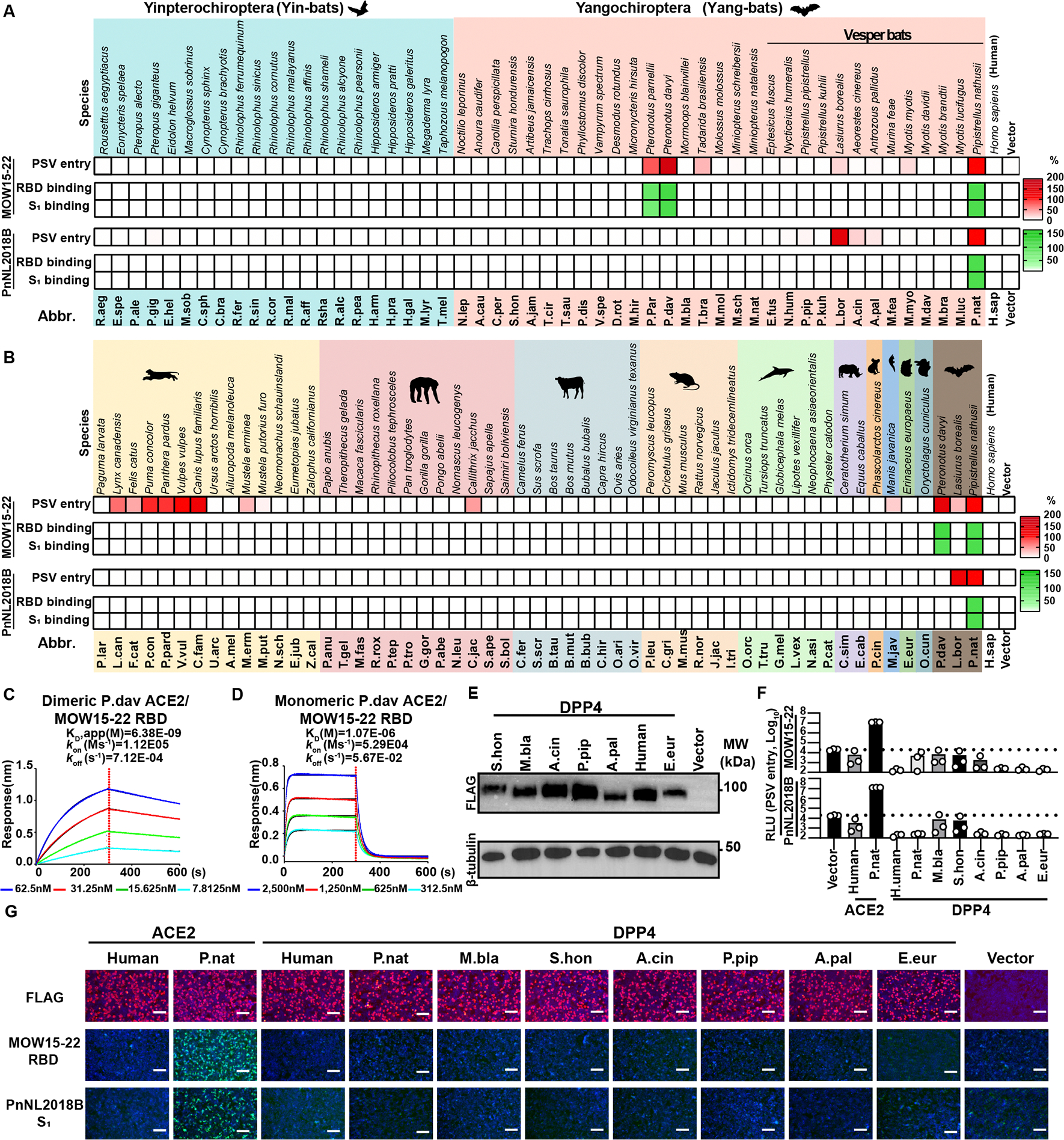
Multi-species ACE2 tropism of MOW15–22 and PnNL2018B. (**A-B**) Heat map representing MOW15–22 PSV entry into and RBD/S_1_ subunit (4 μg/ml) binding to HEK293T cells transiently expressing various ACE2 orthologs from bats (A) or other mammalian species (B). Different mammalian orders are denoted with backgrounds of distinct colors, from left to right: Carnivora, Primates, Artiodactyla, Rodentia, Cetacea, Perissodactyla, Diprotodontia, Pholidota, Erinaceomorpha, Lagomorpha, and Chiroptera. Data are normalized relative to P.nat ACE2 and shown as mean values. Heatmaps are plotted as mean values (n=3 biological replicates). Data are representative of two independent experiments. (**C-D**) BLI analysis of binding kinetics of the dimeric (C) or monomeric (D) P.dav ACE2 ectodomains to biotinylated monomeric MOW15–22 RBD immobilized on SA biosensors. Analysis was conducted with curve-fitting kinetic with global fitting (1:1 binding model) and the fits are shown as black lines. The color keys indicate the concentration of ACE2 used. (**E**) Expression levels of several mammalian DPP4 orthologs in HEK293T cells. (**F**) PSV entry efficiency of MOW15–22 and PnNL2018B in HEK293T cells transiently expressing the indicated receptors. Dashed lines: threshold of the background entry. Data are represented as mean for n=3 biological replicates. (**G**) MOW15–22 and PnNL2018B RBD-hFc binding to HEK293T cells transiently expressing the indicated receptors assessed by immunofluorescence. Scale bars: 100 μm. See also Figure S1–S2.

To explore the potential host range of MOW15–22 and PnNL2018B, we assessed the ability of various ACE2 orthologs transiently transfected in HEK293T cells to promote RBD/S_1_ subunit binding and pseudovirus entry. We used a receptor library comprising 105 ACE2 orthologs from 52 bats and 53 non-bat mammalian species with validated expression (Figure S1A–1B).^24^ MOW15–22 efficiently used three bat ACE2s (P.nat, P.par, P.dav), along with a few others to a lesser extent , while PnNL2018B solely used P.nat and L.bor ACE2 efficiently for entry, indicating host ACE2 specialization during virus evolution (Figure 3A). In contrast to NeoCoV and PDF-2180, MOW15–22 and even more so PnNL2018B had limited tropism for non-bat mammalian ACE2s (Figure 3B), with only a few orthologs from Carnivora and Primates species, such as dog (C.fam) and common marmoset (C.jac) supporting MOW15–22 entry (Figure 3B).

Concurring with the entry data, the human IgG1 Fc domain (hFc)-fused MOW15–22 RBD and S_1_ subunit bound P.nat, P.dav, and P.par ACE2s efficiently, whereas the PnNL2018B RBD and S_1_ subunit interacted strongly with P.nat ACE2 only (Figures 3A–3B). Despite nearly identical ortholog binding profiles between the RBD or S_1_ of both viruses, the latter construct exhibited higher sensitivity for detecting signals from weak binders, such as dog (C.fam) or fox (V.vul) ACE2s (Figure S1C). Consistent with the phenotype observed in Figure 2I–2K, the inclusion of SD1/SD2, but not the NTD, enhanced RBD binding efficiency, enabling the further detection of binding signal with several ACE2 orthologs that can support MOW15–22 PSV entry (Figure S1D). Therefore, we recommend using the S_1_ subunits of these viruses to assess ACE2 binding efficiency and identify receptor candidates.

Notably, two Pteronotus bat ACE2s (P.dav and P.par) but not the additional two Pipistrellus bat orthologs tested (P.pip and P.kuh) were functional receptors, underscoring the importance of specific sequence determinants over phylogenetic relationships of the bat hosts. Using BLI, we observed that dimeric soluble P.dav ACE2 bound to the MOW15–22 RBD with an apparent affinity (K_D_, app) of 6.38 nM whereas monomeric P.dav ACE2 had an affinity (K_D_) of 1.07 μM, outperforming the binding of P.nat ACE2 (Figures 3C–3D). We also tested several DPP4 orthologs from bats, humans, and hedgehogs, and none of them showed any detectable receptor function (Figures 3E–3G).

We observed pseudovirus entry for several ACE2 orthologs for which only weak or no MOW15–22 S_1_ binding could be detected (Figures 3B, S1D, and S2A–2B), as previously described for other coronaviruses.^9,35^ This apparent discrepancy may be attributed to the use of TPCK-treated trypsin to enhance pseudovirus entry with weak receptors and to the multivalent presentation of S trimers on pseudoviruses markedly increasing binding avidity, as compared to dimeric RBD or S_1_-hFc.^36^ Accordingly, L.bor ACE2 promoted pseudovirus entry and membrane fusion in the presence of exogenous trypsin, although no MOW15–22 or PnNL2018B S_1_ binding to this ACE2 was detected (Figure S2C–2F). The addition of exogenous trypsin can thus improve pseudovirus entry assay sensitivity to identify weakly functional ACE2 orthologs.^36^

### Host ACE2 tropism determinants for MOW15–22 and PnNL2018B

**Figure 4.**
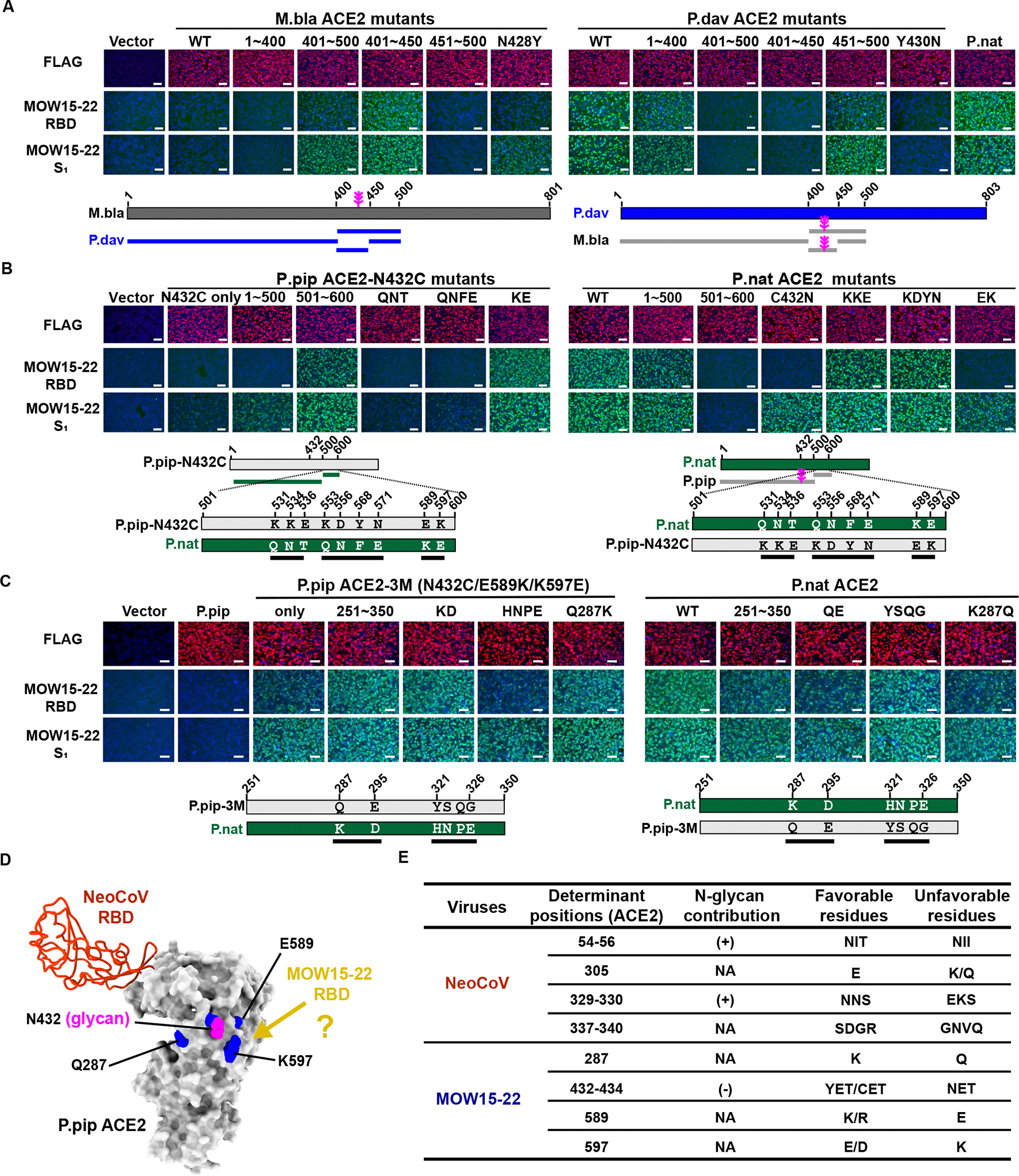
Host ACE2 tropism determinants for MOW15–22 and PnNL2018B. (**A**) MOW15–22 RBD and S_1_ subunit binding to HEK293T cells transiently expressing ACE2 chimeras with sequence swaps between M.bla and P.dav ACE2s analyzed by immunofluorescence. (**B**) MOW15–22 RBD and S_1_ subunit binding to HEK293T cells transiently expressing ACE2 chimeras with sequence swaps between P.pip ACE2-N432C and P.nat ACE2 analyzed by immunofluorescence. (**C**) MOW15–22 RBD and S_1_ subunit binding to HEK293T cells transiently expressing ACE2 chimeras with sequence swaps between P.pip ACE2 N432C/E589K/K597E mutant and P.nat ACE2 analyzed by immunofluorescence. (**D**) Summary of key receptor host range determinants for MOW15–22 and PnNL2018B highlighted in blue on the P.pip ACE2 surface (PDB 7WPO). The N432-glycan is rendered in magenta. (**E**) Summary of ACE2 residues and glycans governing receptor utilization for NeoCoV and MOW15–22. The contribution of N-glycans to receptor binding and representative favorable/unfavorable ACE2 residues are indicated. (**+**): residue promoting binding; (**−**): residue restricting binding; NA: not applicable (not glycosylation site). The P.pip ACE2 residue numbering is shown. Residue positions, N432_P.pipACE2_ glycans (in magenta), and swap strategies are indicated with the schematic below each panel in A, B, and C. Scale bars:100 μm. See also Figure S3–S4.

We previously identified four host range molecular determinants (designated A-D) on the ACE2 receptor for NeoCoV/PDF-2180.^24^ To assess their relevance for species-specific receptor recognition by MOW15–22 and PnNL2018B, we generated P.dav, P.par, P.pip, and L.bor ACE2 mutants harboring changes that would be unfavorable for NeoCoV S-mediated entry. Unexpectedly, none of these alterations markedly affected MOW15–22 S receptor utilization (Figure S3). Moreover, exchanging residues 1–400 between M.bla ACE2 and P.dav ACE2 had no effect on their ability to promote MOW15–22 RBD and S_1_ binding and pseudovirus entry in spite of comprising all of the corresponding residues recognized by NeoCoV (Figure 4A and S4A–4B). Additional sequence swaps led to the identification of residues 400–450 and subsequently the N428_M.blaACE2_ glycosylation site (Y430_P.davACE2_) as critical host range determinants for both MOW15–22 and PnNL2018B, as the presence of this oligosaccharide abolished P.dav ACE2 binding (Figures 4A and S4C–4D). However, the restrictive effect of this glycan is less pronounced on MOW15–22/PnNL2018B S_1_ binding to P.nat ACE2, underscoring possible differences in interactions with different ACE2s (Figures S4E–4F). Furthermore, the N432C mutation, which abrogates the glycosylation site, was insufficient to enable strong binding of the MOW15–22 RBD or S_1_ to P.pip ACE2 without the additional substitutions of residues 500–600 with those of P. nat ACE2 or of residues E589_P.pipACE2_ and K597_P.pipACE2_ to the P. nat ACE2 equivalents (K589 and E597) (Figure 4B and S4G). Consistently, P.nat ACE2 chimera with residues 500–600 replaced by P.pip ACE2 equivalents lost MOW15–22 RBD/S_1_ binding. Furthermore, substitutions at position K287_P.natACE2_ were identified to modulate MOW15–22 and PnNL2018B RBD and S_1_ binding to P.nat ACE2 and P.pip ACE2 (Figures 4C and S4H). Given that the residues identified as governing receptor utilization are spatially close to each other but far from the NeoCoV/PDF-2180 footprint on ACE2, along with the distinct phenotypes of sensitivity to specific ACE2 glycans, we hypothesized that MOW15–22 and PnNL2018B rely on a distinct mode of receptor engagement (Figures 4D–4E).

### Structural basis of MOW15–22 and PnNL2018B utilization of ACE2

**Figure 5.**
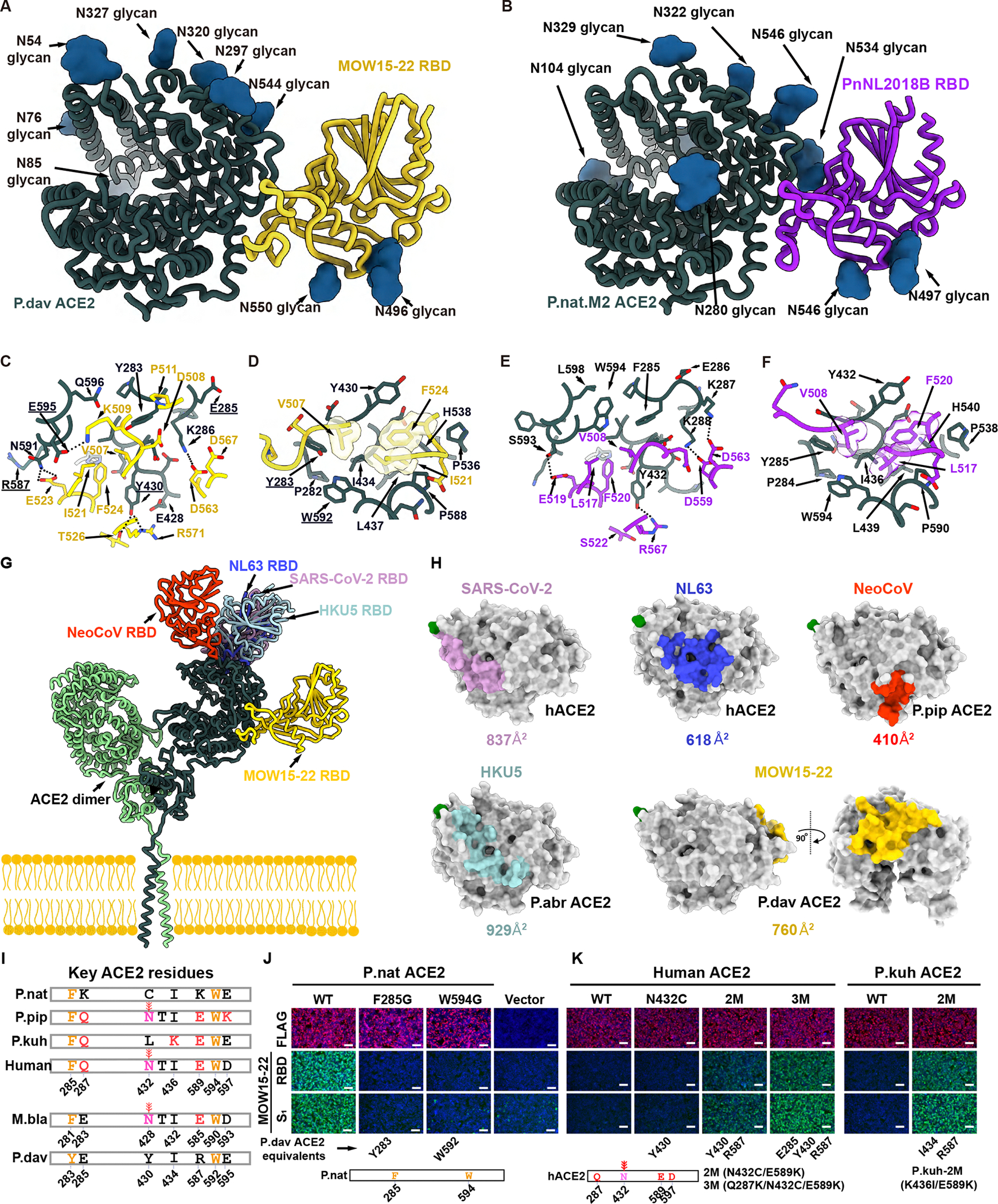
Structural basis for MOW15–22 binding with bat ACE2. (**A-B**) Cryo-EM structure of the MOW15–22 RBD bound to the P.dav ACE2 ectodomain (A) and the PnNL2018B RBD bound to the P.nat.M2 ACE2 ectodomain (B). The ACE2 dimerization domains are omitted for clarity. (**C-F**) Zoomed-in views of key interactions mediating MOW15–22 RBD (yellow) binding to P.dav ACE2 (dark green) (C-D) and PnNL2018B RBD (purple) binding to P.nat.M2 ACE2 (dark green) (E-F). Selected salt bridges and hydrogen bonds are shown as black dotted lines. (**G)** Comparison of the binding modes of the MOW15–22, NeoCoV (PDB 7WPO), SARS-CoV-2/SARS-CoV-1 (PDB 7TN0), NL63 (PDB 3KBH) and HKU5–19s (PDB 9D32) RBDs on bat (not shown for clarity) or hACE2 (PDB 6M1D, B0AT1 not shown for clarity). (**H**) RBD footprints of the five ACE2-using coronaviruses. N-terminus labeled in green. Average areas of the buried interaction interfaces are indicated for the indicated viruses. (**I**) Schematic of critical residues responsible for species-specific receptor recognition in selected ACE2 orthologs. (**J**) P.nat ACE2 mutants with reduced MOW15–22 RBD or S_1_ subunit binding due to unfavorable substitutions to hydrophobic interactions. (**K**) Human or P.kuh ACE2 mutants with improved MOW15–22 RBD or S_1_ subunit binding due to favorable substitutions mapping to the N432-glycosylation site, hydrophobic interactions, or polar interactions. See also Figure S5–S6 and Table S1–S3.

To understand the molecular basis of ACE2 utilization by these recently identified merbecoviruses, we determined a cryo-EM structure of the MOW15–22 RBD bound to the dimeric P.dav ACE2 ectodomain at 2.8Å resolution (Figures 5A and S5A–S5E; Table S1). Two MOW15–22 RBD extensions, spanning residues 505–526 and 563–571, protrude at the distal end of the receptor-binding motif (RBM) to interact with a glycan-free epitope in the ACE2 peptidase domain (Figure 5A; Tables S2–S3). Furthermore, we determined a cryo-EM structure of the PnNL2018B RBD bound to the dimeric P.nat.M2 ACE2 chimera ectodomain (described below) at 2.4Å resolution (Figures 5B and S5F–S5J; Table S1). The PnNL2018B RBD uses its two RBM extensions to recognize a very similar ACE2 surface, as that of MOW15–22, albeit involving the P.nat M2 ACE2 N534 glycan which is absent in P.dav ACE2 due to the N534_P.nat.M2ACE2_ to Q532_P.davACE2_ substitution (Figure 5B; Table S2 and Table S3).

The MOW15–22 and PnNL2018B RBDs largely adopt a canonical merbecovirus architecture comprising a core folded as a five-stranded antiparallel β-sheet with two α-helices and a four-stranded antiparallel β-sheet RBM with one α-helix (Figures 5A–5B). However, these two RBDs are set apart from other merbecoviruses due to their elongated and twisted distal RBM extensions which form the ACE2-binding surface, one of them being stabilized by the MOW15–22 C506-C520 disulfide (PnNL2018B C507-C516), and to the elongated α-helix flanking the RBM on the opposite side. Furthermore, the MOW15–22 and PnNL2018B RBDs harbor two N-linked glycans protruding from the base of the RBM that are unique to this clade of merbecoviruses (Figure 5A–5B).

The MOW15–22 RBD/P.dav ACE2 and PnNL2018B RBD/Pnat.M2 ACE2 interfaces respectively bury an average of 760 Å^2^ and 610 Å^2^ at their protein interfaces and select key interactions include (**i**) salt bridges between K286_P.davACE2_ (K288_P.nat.M2ACE2_) and D563_MOW15–22_ (D559_PnNL2018B_) as well as between K287_P.nat.M2ACE2_ and D563_PnNL2018B_; (**ii**) hydrogen-bonding between Y430_P.davACE2_ (Y432_P.nat.M2ACE2_) and R571_MOW15–22_ (R567_PnNL2018B_); (**iii**) hydrogen-bonding between N591_P.davACE2_ (S593_P.nat.M2ACE2_) and E523_MOW15–22_ (E519_PnNL2018B_); (**iv**) a salt bridge between E595_P.davACE2_ and K509_MOW15–22_; (**v**) extensive hydrophobic interactions mediated by V507_MOW15–22_ (V508_PnNL2018B_), I521_MOW15–22_ (L517_PnNL2018B_), and F524_MOW15–22_ (F520_PnNL2018B_) with ACE2. The distinct lengths and local structures of the RBM insertion 1 likely explain the enhanced binding affinity (and therefore breadth) for several ACE2 orthologs of MOW15–22 relative to PnNL2018B. Indeed, RBM insertion 1 accounts for two-thirds of the difference in buried surface area between the two structures. Integrating these findings with our mutagenesis data provides a molecular framework to understand the identified determinants of host receptor species tropism (Fig 4C–F).

Strikingly, the MOW15–22 and PnNL2018B RBDs recognize ACE2 at a site located more than 45Å away from the sites recognized by SARS-CoV-2, NL63, NeoCoV, or HKU5 (Figures 5G–5H). This suggests that ACE2-utilization was acquired multiple times independently among these coronaviruses, including at least three times for merbecoviruses. Our data explain that the absence of a glycan at P.dav ACE2 Y430 (P.nat ACE2 C432), which is otherwise conserved in several other ACE2 orthologs (e.g., hACE2/P.pip N432 and M.bla N428 glycans), removes a steric barrier which would impede MOW15–22 and PnNL2018 RBD engagement, concurring with the lack of binding observed to a P.dav Y430N glycan knockin mutant (Figure 4A). Accordingly, ACE2 orthologs lacking this glycan (e.g. P.par, C.jac, and L.bor ACE2s) support efficient MOW15–22 or PnNL2018B S pseudovirus entry into cells (Figures 3A–3B), and these two viruses are therefore not expected to acquire efficient hACE2 utilization without major adaptations.

To further assess the contribution of the identified receptor binding determinants, we analyzed the impact of residue changes at key positions of P.nat, P.pip, human, P.kuh, and marmoset (C.jac) orthologs on binding (Figures 5I–K and S6A–D). The P.nat ACE2 F285G and W594G interface mutants had severely dampened binding to MOW15–22 and PnNL2018B, whereas three hACE2 residue substitutions (Q287K/N432C/E589K) and two P.kuh ACE2 residue substitutions (K436I/E589K) promoted detectable binding to MOW15–22 and PnNL2018B (Figures 5J, 5K and S6A–6B). The Q287K ACE2 substitution enables the formation of a salt bridge with D563_PnNL2018B_ (and likely D567_MOW15–22_) and the E589K ACE2 substitution likely relieves electrostatic repulsion with E523_MOW15–22_ or E519_PnNL2018B_. C.jac ACE2 S432N glycan knock-in mutation abolished the receptor function of the sole primate ortholog supporting entry, further underscoring the key role of this oligosaccharide as a tropism barrier. Conversely, the C.jac ACE2 Q287K and Q598L point mutations (to promote additional interactions) markedly improved binding affinity to both MOW15–22 and PnNL2018B (Figure S6C–6D). Concurring with the structural data, introducing unfavorable changes to the MOW15–22 RBM, either by reducing interaction or abolishing the C506-C520 disulfide, significantly reduced RBD binding and pseudovirus entry in P.nat or P.dav ACE2-expressing HEK293T cells (Figure S6E–6G).

### Characterization and inhibition of MOW15–22 and PnNL2018B ACE2-mediated entry

**Figure 6.**
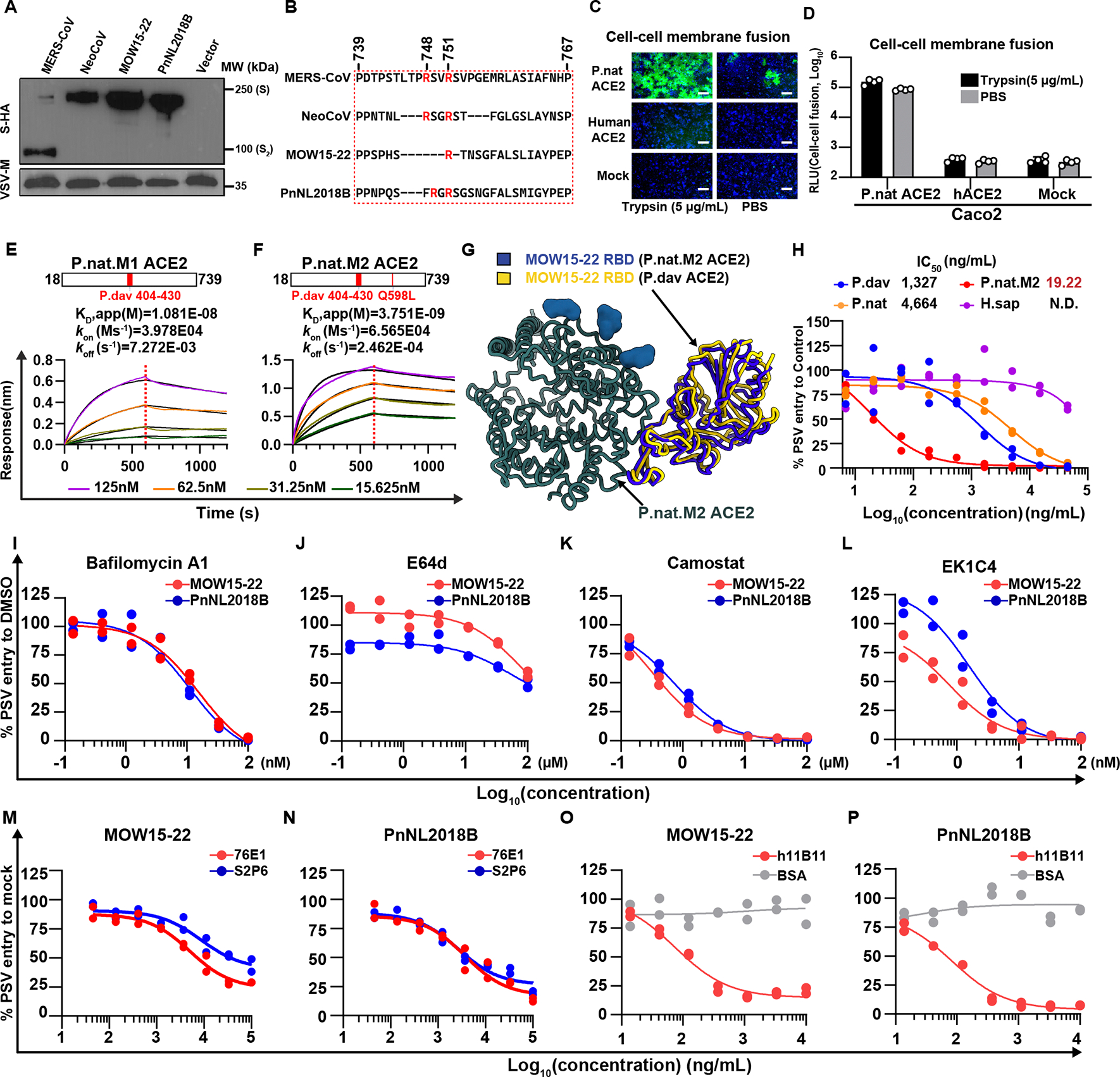
Characterization and inhibition of MOW15–22 and PnNL2018B ACE2-mediated entry. (**A**) Quantification of S glycoprotein incorporation in VSV pseudotypes analyzed by Western blot detecting the C-terminal-fused HA tags. VSV-M was used as a loading control. (**B**) Sequence analysis of S glycoprotein S_1_/S_2_ junction (dashed box) from the indicated viruses, with arginine highlighted in red. (**C-D**) MOW15–22 S-mediated cell-cell fusion using Caco2 cells stably expressing P.nat ACE2 upon addition of TPCK-treated trypsin. The signal resulting from the reconstitution of split sfGFP (C) or RLuc (D) is indicated. Data are represented as mean ± SD for n=3 biological replicates in D. Scale bars:200 μm. (**E-F**) BLI analyses of binding kinetics of soluble dimeric ACE2 ectodomains from P.nat.M1 ACE2 (E) and P.nat.M2 ACE2 (F) carrying P.dav ACE2 equivalent residues (red) to immobilized MOW15–22 RBD-hFc. Global fitting to the data using a 1:1 binding model is shown in black. (**G**) Superimposition of the cryoEM structures of the MOW15–22 RBD (gold) in complex with P.dav ACE2 (not shown for clarity) and of the MOW15–22 RBD (blue) bound to P.nat.M2 ACE2 (dark green) superimposed based on the ACE2 peptidase domain. (**H**) Dose-dependent inhibition of MOW15–22 pseudovirus entry mediated by soluble P.dav, P.nat, P.nat.M2 and human ACE2s in Caco2 cells stably expressing P.nat ACE2. IC_50_ values are indicated. N.D.: Not detectable. (**I-L**) Inhibitory activities of small compounds or peptide inhibitors against MOW15–22 and PnNL2018B pseudoviruses using Caco2 cells stably expressing the hACE2–3M mutant (Q287K/N432C/E589K). Endosomal acidification inhibitor bafilomycin A1 (Baf-A1) (I), cathepsin L inhibitor E64d (J), TMPRSS2 inhibitor camostat (K), S_2_-targeting HR2-derived peptide fusion inhibitor EK1C4 (L). (**M-P**) Dose-dependent inhibition of MOW15–22 and PnNL2018B pseudoviruses by S2P6 and 76E1 (M-N), or h11B11 (O-P) in the Caco2-hACE2–3M cells. For I-P, data are representative of two independent experiments with similar results (n=2 biological replicates). See also Figure S7 and Table S1.

MERS-CoV efficiently utilizes endogenous host proteases, such as furin and TMPRSS2, for cellular entry.^37^ MERS-CoV S harbors a polybasic (furin) cleavage site at the junction between the S_1_/S_2_ subunits, leading to proteolytic processing during biogenesis.^38^ Conversely, MOW15–22 and PnNL2018B S glycoproteins lack an S_1_/S_2_ furin cleavage site and remain uncleaved upon incorporation into VSV pseudovirus particles, suggesting distinct protease requirements for cellular entry compared to MERS-CoV (Figures 6A–B). Accordingly, MOW15–22 S glycoproteins are highly dependent on the addition of exogenous trypsin to promote cell-cell fusion, indicating a potential reliance on endosomal pathways for cellular entry in Caco2 cells stably expressing P.nat ACE2, which endogenously express TMPRSS2 at their surface (Figures 6C–6D).^39^

To compete viral entry using soluble ACE2, we designed P.nat ACE2 recombinant ectodomain chimeras carrying P.dav ACE2 residues 404–430 without (P.nat.M1 ACE2) or with the Q598L substitution (P.nat.M2 ACE2) which exhibited enhanced binding affinity for both MOW15–22 and PnNL2018B RBD-Fc constructs relative to wildtype P.nat ACE2 (Figures 6E–6F and S7A–C).^9^ Cryo-EM analysis of the complex between the MOW15–22 RBD and P.nat.M2 ACE2 confirmed retention of a native binding mode, as is the case in the PnNL2018B RBD-bound structure described above, and explains the observed enhanced binding (Figures 6G and S7D–S7G; Table S1). We observed concentration-dependent soluble P.nat.M2 ACE2-mediated inhibition of MOW15–22 and PnNL2018B S pseudoviruses with a half-maximal inhibitory concentration (IC_50_) of 19.22 ng/ml, outperforming wild-type P.nat and P.dav ACE2s (Figure 6H), further confirming the potential of these ACE2 mutants for developing entry inhibitors.

To identify other countermeasures against these divergent merbecoviruses, we assessed the ability of entry inhibitors, monoclonal antibodies, nanobodies, and peptide fusion inhibitors to block S-mediated entry in Caco2 cells stably expressing the functional hACE2 mutants harboring the Q287K, N432C, and E589K mutations (hACE2–3M) (Figures 6I–6R and S7H–S7L). Endosomal entry inhibitors, such as Bafilomycin A and E64d, as well as the TMPRSS2 inhibitor camostat inhibited pseudovirus entry in a dose-dependent manner (Figures 6I–6L).^39,40^ These results suggest that the proteolytic activation of MOW15–22 S and PnNL2018B S can be mediated by either cell surface TMPRSS2 or endosomal cysteine proteases such as Cathepsin B/L in Caco2 cells. Whereas MERS-CoV RBD-directed nanobodies were ineffective against MOW15–22 and PnNL2018B S pseudoviruses (Figure S11A),^41,42^ broadly neutralizing S_2_ subunit-directed monoclonal antibodies targeting the stem helix (S2P6) or the fusion peptide/S_2_’ cleavage site (76E1) retained activity, consistent with the marked divergence of their RBDs and conservation of the targeted S_2_ epitopes (Figures 6M–6N and S7I–K).^43,44^ Furthermore, the EK1C4 HR2-derived peptide and the hACE2-targeting antibody h11B11 efficiently neutralized the entry of MOW15–22 and PnNL2018B pseudoviruses (Figures 6O, 6P, and S7L).^45–48^ Collectively, these data demonstrate that neutralizing antibodies targeting the fusion machinery (S_2_ subunit) or the host receptor prevent ACE2-mediated entry and could be potentially used as countermeasures against these viruses.

### MOW15–22 and PnNL2018B S-mediated propagation supported by ACE2

**Figure 7.**
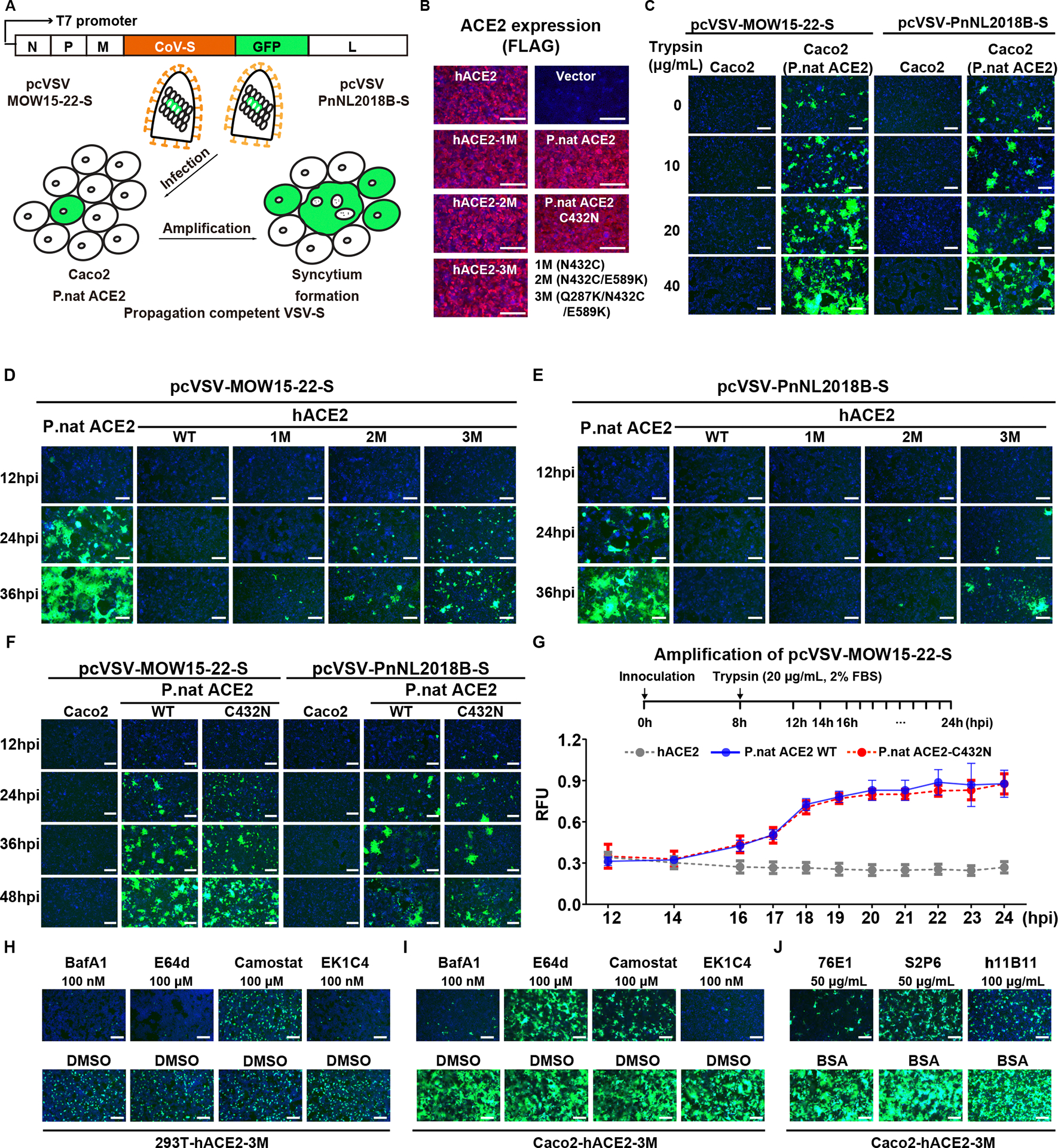
MOW15–22 and PnNL2018B S-mediated propagation supported by ACE2. (**A**) Genetic organization and amplification of pcVSV-MOW15–22-S and pcVSV-PnNL2018B-S. (**B**) Immunofluorescence analyzing the expression of P.nat and human ACE2 mutants stably expressed in Caco2 cells. (**C**) GFP signal from TPCK-trypsin-enhanced amplification of pcVSV-MOW15–22-S and pcVSV-PnNL2018B-S in Caco2 cells with or without the expression of P.nat ACE2 at 24hpi. (**D-E**) Propagation of pcVSV-MOW15–22-S (D) and pcVSV-PnNL2018B-S (E) in Caco2 cells stably expressing hACE2 carrying N432C (1M), N432C/E589K (2M), Q287K/N432C/E589K (3M) mutants, with P.nat ACE2 as a positive control. (**F**) Representative fluorescence images of pcVSV-MOW15–22-S and pcVSV-PnNL2018B-S amplification in Caco2 cells expressing P.nat ACE2 or P.nat ACE2-C432N mutants at 12, 24 and 36-hour post-infection (hpi). (**G**) pcVSV-MOW15–22-S propagation kinetics in Caco2 cells expressing P.nat ACE2 or P.nat ACE2-C432N mutants at the indicated time points. Data are represented as mean ± SD for n=3 biological replicates. RFU: relative fluorescence unit. TPCK-treated trypsin was present in the cells at 20 μg/mL (DMEM+2%FBS) in D, E, F, and G. (**H-J**) pcVSV-MOW15–22-S propagation in the presence of indicated inhibitors in 293T-hACE2–3M cells (H), Caco2-hACE2–3M cells (I), or indicated antibodies in Caco2-hACE2–3M cells (J). BSA: Bovine serum albumin, 50 μg/ml. hpi: hours post-infection. Scale bars:200 μm. See also Figure S7.

We utilized a reverse genetic system to create propagation-competent VSV-Spike recombinant viruses (pcVSV-S) as surrogates to characterize the MOW15–22 and PnNL2018B S glycoprotein-mediated propagation supported by different ACE2s. The VSV-G genes in these recombinant viruses were replaced with the MOW15–22 or the PnNL2018B S glycoprotein gene, with an additional GFP gene for readout (Figures 7A–7B). After rescuing the viruses, we observed a dose-dependent trypsin enhancement of amplification in Caco2 cells expressing P.nat ACE2, as evidenced by syncytia formation (Figure 7C). Although viral propagation was not observed in Caco2 cells overexpressing hACE2, abrogation of the N432 glycan (via the N432C mutation) resulted in detectable amplification of pcVSV-MOW15–22-S. Furthermore, introducing the E589K or the Q287K/E589K mutations in the hACE2 N432C background further enhanced pcVSV-MOW15–22-S amplification efficiency (Figure 7D) and enabled detection of pcVSV-PnNL2018B-S amplification (Figure 7E), concurring with our binding data (Figure 5H). Despite the restriction on viral recognition imposed by the hACE2 N432 glycan, the two viruses could nevertheless utilize P.nat ACE2 harboring the C432N (glycan knockin) mutation, as demonstrated by observed viral propagation and S_1_ subunit binding (Figures 7F–7G and S4E–F). The phenotype indicates that these viruses can overcome the steric tropism barrier imposed by the N432 glycan, possibly through compensatory interactions in other ACE2 regions. Consistent with the single-round PSV entry data (Figure 6 I–P), Bafilomycin A1, EK1C4, S2P6, 76E1, and h11B11 inhibited pcVSV-MOW15–22-S amplification (Figure 7H–J and S7M). Moreover, pcVSV-MOW15–22-S inhibition occurred in Caco2-hACE2–3M cells for camostat and in HEK293T-hACE2–3M for E64d, concurring with the presence or absence of TMPRSS2 in these two cell lines, respectively.

## DISCUSSION

Coronaviruses exhibit remarkable variations in RBD sequences, resulting in diverse receptor usage across different viruses.^49^ While coronaviruses within the same genus or subgenus typically share similar RBD core structures, RBM variability can result in entirely different receptor usage.^9,50,51^ Conversely, phylogenetically distant coronaviruses can convergently evolve to engage the same receptor during evolution. For example, APN is a receptor shared by several α-coronaviruses and δ-coronaviruses,^51–53^ whereas ACE2 serves as a functional receptor for three viral subgenera that belong to α-coronaviruses (*Setracoviruses*) and β-coronaviruses (*Sarbecoviruses* and *Merbecoviruses*) .^21,54–56^

The discovery of ACE2 usage in European bat MERSr-CoVs expands the diversity of ACE2-using merbecoviruses beyond NeoCoV and PDF-2180 and reveals that coronaviruses belonging to the same subgenus or even species (e.g. MOW15–22 and NeoCoV) can engage entirely distinct surfaces of a same receptor (e.g. P.dav ACE2). This study emphasizes the need to characterize receptor usage and binding modes experimentally, instead of solely relying on *in silico* predictions. In this study, we employed two functional assays (antigen binding and PSV entry) to assess the MOW15–22 and PnNL2018B ACE2 receptor tropism. These assays evaluate receptor function from different aspects and cross-validate each other. PSV entry assays are more sensitive for identifying weak receptors, probably due to the TPCK-trypsin treatment and the increased avidity resulting from the multivalent presentation of S trimers on PSV, whereas binding assays are more useful for evaluating receptor fitness, as demonstrated in our binding determinant mapping assays. Notably, we observed that both MOW15–22 and PnNL2018B S_1_-hFc exhibited higher efficiency in binding to weak receptors compared to their corresponding RBD-hFc constructs, and the improvement is attributed to the inclusion of SD1/SD2 subdomains which may enhance biochemical stability or binding avidity by altering the distance and geometry between the two RBDs. The observation of enhanced binding with S_1_ versus RBD does not impact our conclusions regarding host tropism, as these S_1_ binders are all orthologs capable of facilitating PSV entry.

Our data further support the hypothesis that ACE2 usage was convergently opted by different coronaviruses with remarkable differences in their RBD or RBM sequences and structures. Coronavirus RBM insertions/deletions (indels) may play a crucial role in receptor switch and subsequent adaptation,^57^ as demonstrated by the presence of two RBM insertions and a disulfide bond specific to MOW15–22 and PnNL2018B (Figure 1E). MOW15–22 is possibly further along this evolutionary trajectory of adapting to ACE2 as it carries a 5-amino acid longer insertion 1 than PnNL2018B, enabling more extensive interactions with ACE2 and allowing it to engage with several non-bat mammalian ACE2 orthologs. Furthermore, we show in our companion paper that HKU5 utilizes its host P.abr ACE2, along with a few other orthologs, via a fifth ACE2 binding mode.^33^ Notably, these ACE2-using merbecoviruses with distinct binding modes putatively evolved in three different Old World continents, suggesting the convergent evolution of ACE2 adaptation and the importance of investigating the global prevalence and distribution of ACE2-using merbecoviruses. This ACE2 preference likely confers certain evolutionary advantages in transmission, as exemplified by the highly transmissible SARS-CoV-2 omicron variants.^58,59^ However, a recent study reported that PnNL2180B (PN-βCoV) primarily exhibits intestinal tropism in its natural bat host, suggesting a potential fecal-oral route used by these viruses in bats.^28^ Given that airborne transmission is the major route of all known ACE2-using human coronaviruses, it is important to investigate whether tissue tropism and transmission route changes when ACE2-using viruses jump from bats to other mammals, especially humans.

Interestingly, ACE2 glycans can play contrasting roles in host range determination of ACE2-using merbecoviruses. For example, unlike the positive role of ACE2 glycans (e.g. N54 and N329 glycans) in NeoCoV and PDF-2180 recognition^9^, the N432 glycan in the interaction interface restricted efficient MOW15–22 and PnNL2018B binding, contributing to narrower ACE2 tropism, particularly for PnNL2018B for which we only detected binding to its host P. nat ACE2^9^. Common marmoset (C.jac) ACE2, which is the only primate ortholog supporting MOW15–22 S-mediated entry, lacks an N432 glycan which is otherwise conserved in other primate ACE2s, explaining its distinct phenotypes. This specialized adaptation to host ACE2s lacking an N432-glycan is expected to prevent ACE2 utilization from many other species, including humans. However, the efficient amplification of pcVSV-MOW15–22-S and pcVSV-PnNL2018B-S supported by a P.nat ACE2 mutant (C432N) harboring an N432 glycan suggests putative evolutionary pathways by which these viruses could overcome this obstacle, underscoring the usefulness of the countermeasures identified here. Notably, the VM314 viral sequence, identified in samples collected in the Netherlands in 2008 based on an RNA-dependent RNA polymerase (RdRp) gene fragment that is phylogenetically close to MOW15–22 and PnNL2018B, may already be adapted to its host (P.pip) ACE2 carrying an N432 glycan. Future studies should be conducted to obtain the corresponding S glycoprotein sequences and to verify whether these viruses recognize ACE2 in a similar way to MOW15–22 and PnNL2018B.^60^

Previous studies proposed that the emergence of the DPP4-using MERS-CoV may be associated with a receptor switch from ACE2 to DPP4 through recombination.^9^ This could have occurred between an ACE2-using bat MERSr-CoV and a yet-to-be-identified DPP4-using merbecovirus, such as HKU4 or CoV-422.^15,61^ We note that CoV-422 was also suggested to have originated from recombinations leading to receptor switch^15^. Additionally, it has been suggested that ACE2-using merbecoviruses might have arisen through recombination between ancestral viruses of bats and hedgehogs.^62^ However, no evidence of ACE2 usage for coronaviruses HKU31 or other EriCoVs has been reported to date.^9,63,64^ These observations raise intriguing and important questions regarding the evolution trajectory of receptor usages of merbecoviruses and whether ACE2 receptor usage is the more ancestral trait for merbecoviruses than DPP4.

Our study profoundly changes our understanding of ACE2-using merbecoviruses by identifying and characterizing two bat merbecoviruses with previously unknown ACE2 binding modes, involving binding sites located >45Å away from that recognized by any other coronaviruses. This discovery also underscores the likelihood of the existence of other yet-to-be-discovered ACE2-using merbecoviruses, further expanding the diversity and geographic distribution of these viruses with spillover potential. Although the pathogenicity and transmission abilities of these viruses remain unclear, enhanced surveillance along with identification of viral inhibitors are warranted to proactively detect and prepare for potential zoonosis caused by ACE2-using merbecoviruses.

### Limitations of the study

A limitation of this study is the lack of infection data using authentic coronaviruses, as no MOW15–22 or PnNL2018B isolates are available to date. Authentic MOW15–22 and PnNL2018B may exhibit subtle differences in sensitivity to inhibitors compared to the VSV-based pseudotypes or propagation-competent VSV chimeras used in this study. Furthermore, although we identified ACE2 orthologs supporting MOW15–22 or PnNL2018B S-mediated entry from species not known to be hosts for these two viruses, further studies are needed to determine their susceptibility to infection, as other host factors responsible for proteolytic activation, antiviral immunity, and virus replication participate in determining host range. Future studies testing authentic virus infection *in vivo* may provide additional information crucial for assessing their zoonotic risks.

### RESOURCE AVAILABILITY

#### Lead Contact

Further information and requests for resources and reagents should be directed to and will be fulfilled by the lead contact, Huan Yan (严欢) (huanyan@whu.edu.cn)

#### Materials Availability

All plasmids generated in this study are available with a completed Materials Transfer Agreement. Correspondence and requests for materials can be addressed to the lead contact.

#### Data and Code Availability

- The cryo-EM structures have been deposited to the electron microscopy data bank and protein data bank with accession numbers EMD-45253, PDB-9C6O (P.dav ACE2-bound MOW15–22 RBD), EMD-46691, PDB-9DAK (P.nat.M2 ACE2-bound PnNL2018B RBD) and EMD-60483, PDB-8ZUF (P.nat.M2 ACE2-bound MOW15–22 RBD). Original western blot images, P.nat ACE2- and P.nat DPP4-coding sequences have been deposited in Mendeley with DOIs and accession numbers listed in the key resources table.
- This paper does not report original code.
- Any additional information required to reanalyze the data reported in this paper is available from the lead contact upon request.

## STAR ★ METHODS

### EXPERIMENTAL MODEL AND STUDY PARTICIPANT DETAILS

#### Cell lines and culture conditions

HEK293T (CRL-3216), HEK293T (ATCC, CRL-11268), Caco2 (HTB-37), BHK21 (CCL-10), and I1-Hybridoma (CRL-2700) cell lines were obtained from the American Type Culture Collection (ATCC). Expi293F (A14527) cells were obtained from Thermo Fisher Scientific. These cells were maintained in Dulbecco’s Modified Eagle Medium (DMEM, Monad, China) supplemented with 1% PS (Penicillin/Streptomycin) and 10% Fetal Bovine Serum (FBS). The I1-Hybridoma cell line, which produces a neutralizing antibody targeting the VSV glycoprotein (VSV-G), was cultured in Minimum Essential Medium (MEM) with Earles’s balances salts, 2.0 mM of L-glutamine (Gibico), and 10% FBS. All cell lines were cultured at 37°C with 5% CO_2_ and routinely passaged every 2–3 days. HEK293T or Caco2 stable cell lines overexpressing various receptors were generated using lentivirus transduction and antibiotic selection. The stable cells based on HEK293T or Caco2 were selected and maintained in the growth medium with puromycin (1 μg /ml).

### METHOD DETAILS

#### Plasmids and vectors

Plasmids expressing wild-type (WT) or mutated bat and non-bat mammalian ACE2 orthologs ^24,35^ were constructed by inserting human codon-optimized sequences with/without specific mutations into a lentiviral transfer vector (pLVX-EF1a-Puro, Genewiz) with C-terminus 3×FLAG tags (DYKDHD-G-DYKDHD-I-DYKDDDDK) and single FLAG tags (DYKDDDDK) for non-bat mammalian ACE2 orthologs^24^. P.nat ACE2 and DPP4 coding sequences are retrieved from the genome of *Pipistrellus nathusii* (GCA_963693515.1), P.nat ACE2, P.nat.M1, P.nat.M2 and P.nat DPP4 coding sequences have been deposited at Mendeley with DOI listed in the key resources table^31^. For pseudovirus production, human codon-optimized spike sequences of MOW15–22 (USL83011.1), PnNL2018B (WDE20340.1), MERS-CoV (YP_009047204.1), HKU4 (AWH65899), NeoCoV (AGY29650.2) and HKU31 (QGA70692.1) were cloned into the pCAGGS vector with C-terminal deletions (residues 13–15) for improving the pseudovirus assembly efficiency. The DNA fragments for cloning ACE2 chimera or mutants were generated by overlap extension PCR or gene synthesis and verified by commercial DNA sequencing. For the expression of recombinant CoVs RBD-hFc fusion proteins, plasmids were constructed by inserting NeoCoV RBD (residues 380–585), MOW15–22 RBD (residues 360–610aa), PnNL2018B RBD (residues 361–606) coding sequences into the pCAGGS vector containing an N-terminal CD5 secretion signal peptide (MPMGSLQPLATLYLLGMLVASVL) and C-terminal hFc-twin-strep tandem tags for purification and detection. Plasmids expressing soluble ACE2 ectodomain proteins were generated by inserting sequences from hACE2 (residues 18–740) P.dav ACE2 (residues 18–738), and P.nat ACE2 (residues 18–739) into pCAGGS vector, with an N-terminal CD5 secretion signal peptide and a C-terminal twin-strep-3×FLAG tag (WSHPQFEKGGGSGGGSGGSAWSHPQFEK-GGGRSDYKDHDGDYKDHDIDYKDDDDK)^9^. The construct for expressing soluble P.nat.M1 ACE2 was generated by overlapping PCR to replace residues of 404–430 with P.dav ACE2 corresponding sequences. The P.nat.M2 ACE2 was generated by further introducing a Q598L point mutation to P.nat.M1 ACE2.

For BLI shown in Fig 2–3 and for cryo-EM analysis of the P.dav ACE2-bound MOW15–22 RBD and of the P.nat.M2 ACE2-bound PnNL2018B RBD, the P.dav, P.nat, and P.nat.M2 ACE2 ectodomain dimer (residues 17–728 for P.dav and residues 17–729 for P.nat) constructs harbor an N-terminal signal peptide (MPMGSLQPLATLYLLGMLVASVL) and a C-terminal eight-residue flexible linker, an avi tag, followed by an octa-histidine tag. Dimeric ACE2 ectodomain constructs were codon-optimized, synthesized, and inserted by Genscript into the pcDNA3.1(+) vector for the P.dav construct and pcDNA.3.4 vector for the P.nat constructs. The P.dav and P.nat ACE2 ectodomain monomers (residues 17–613 for P.dav and 17–614 for P.nat) constructs harbor an N-terminal signal peptide (MGILPSPGMPALLSLVSLLSVLLMGCV) and a C-terminal two-residue linker, an avi tag, followed by an octa-histidine tag. Monomeric ACE2 constructs were codon-optimized, synthesized, and inserted into the pcDNA.3.4 vector by Genscript. The MOW15–22 and PnNl2018B RBDs (encoding S residues 360–611 for MOW15–22 and 361–607 for PnNl2018B) constructs harbor an N-terminal signal peptide (MGILPSPGMPALLSLVSLLSVLLMGCVAETG), C-terminal eight-residue flexible linker, an avi tag, followed by an octa-histidine tag. RBD constructs were codon-optimized, synthesized, and inserted into the pcDNA3.1(+) vector by Genscript.

#### Protein expression and purification

HEK293T cells were transfected with corresponding plasmids using GeneTwin reagent (Biomed, TG101–01). Subsequently, the culture medium of the transfected cells was replenished with the SMM 293-TII Expression Medium (Sino Biological, M293TII) 4–6 hours post-transfection, and the protein-containing supernatant was collected every three days for 2–3 batches. Antibodies and recombinant RBD-hFc proteins were purified using Pierce Protein A/G Plus Agarose (Thermo Scientific, 20424). In general, Fc-containing proteins were enriched by the agarose, washed with wash buffer (100 mM Tris/HCl, pH 8.0, 150 mM NaCl, 1 mM EDTA), eluted using the Glycine buffer (100 mM in H_2_O, pH 3.0), and immediately neutralized with 1/10 volume of 1M Tris-HCI, pH 8.0 (15568025, Thermo Scientific). Proteins with twin-strep tag were purified using Strep-Tactin XT 4Flow high-capacity resin (IBA, 2–5030-002), washed by wash buffer (100 mM Tris/HCl, pH 8.0, 150 mM NaCl, 1 mM EDTA), and then eluted with buffer BXT (100 mM Tris/HCl, pH 8.0, 150 mM NaCl, 1 mM EDTA, 50 mM biotin). All eluted proteins were concentrated using Ultrafiltration tubes, buffer-changed to PBS, and stored at −80°C. Protein concentrations were determined by the Omni-Easy Instant BCA Protein Assay Kit (Epizyme, ZJ102).

#### Antigen-hFc live-cell binding assay

RBD- or S_1_-hFc live-cell binding assays were conducted following a previously described protocol^35^. The coronavirus RBD- or S_1_-hFc recombinant proteins were diluted in DMEM at indicated concentrations and incubated with HEK293T cells transiently expressing different ACE2 for 30 minutes at 37°C at 36 hours post-transfection. Subsequently, cells were washed once with Hanks’ Balanced Salt Solution (HBSS) and incubated with 1 μg/mL of Alexa Fluor 488-conjugated goat anti-human IgG (Thermo Fisher Scientific; A11013) diluted in HBSS/1% BSA for 1 hour at 37°C. After another round of washing with HBSS, the cell nuclei were stained with Hoechst 33342 (1:10,000 dilution in HBSS) for 30 minutes at 37°C. The images were captured using a fluorescence microscope (MI52-N). The relative fluorescence intensities (RFUs) of the stained cells were determined by a Varioskan LUX Multi-well Luminometer (Thermo Scientific). The heatmap presentations in Figures 3A and 3B were plotted based on RFUs subtracted with background RLUs in cells without ACE2 expression. Color ranges and thresholds were adjusted to show binding efficiencies consistent with the fluorescence images captured by the microscope.

#### Flow cytometry

To analyze S_1_-hFc binding through flow cytometry, HEK293T cells transiently expressing the indicated ACE2 orthologs were detached with 5 mM EDTA/PBS at 36 hours post-transfection. The cells were washed twice with cold PBS and incubated with MOW15–22 or PnNL2018B S_1_-hFc proteins at 2 μg/mL concentrations at 4°C for 30 minutes. Subsequently, cells were incubated with Alexa Fluor 488-conjugated goat anti-human IgG to stain the RBD (Thermo Fisher Scientific; A11013) at 4°C for 1 hour. Afterward, cells were fixed with 4% PFA, permeabilized with 0.25% Triton X-100, blocked with 1% BSA/PBS at 4°C, and then incubated with mouse antibody M2 (Sigma-Aldrich, F1804) diluted in PBS/1% BSA for 1 hour at 4°C, followed by incubation with Alexa Fluor 594-conjugated goat anti-mouse IgG (Thermo Fisher Scientific; A32728) diluted in 1% BSA/PBS for 1 hour at 4°C. For all samples, 10,000 ACE2-expressing live cells (gated based on SSC/FSC and FLAG-fluorescence intensity and SSC/FSC) were analyzed using a CytoFLEX Flow Cytometer (Beckman).

#### Biolayer interferometry (BLI) binding assay

For dimeric P.nat, P.nat.M1, and P.nat.M2 ACE2 ectodomain proteins binding to immobilized MOW15–22 RBD-hFc (Figure 6E–6F) or PnNL2018B S_1_-hFc (Figure S9), recombinant hFc containing proteins were diluted to 20 μg/mL and immobilized on Protein A (ProA) biosensors (ForteBio, 18–5010), which were then incubated with soluble dimeric bat ACE2-ectodomain proteins, which were two-fold serial-diluted in kinetic buffer (PBST), starting from 1,000 nM. A well incubated with kinetic buffer (PBST) only serves as a background control. Protein binding kinetics was assessed using BLI assays with the Octet RED96 instrument (Molecular Devices) at 25°C, and shaking at 1000 rpm. The kinetic parameters and the apparent binding affinities (due to ACE2 dimerization) were analyzed using Octet Data Analysis software 12.2.0.20 with global curve fitting using a 1:1 binding model.

BLI analysis of dimeric or monomeric P.nat or P.dav ACE2 ectodomains binding to the MOW15–22 or PnNL2018B RBD (Figures 2F–2I and Figures 3C–3D) was performed at 30°C and shaking at 1,000 rpm. The biotinylated monomeric MOW-15–22 or PnNL2018B RBDs were diluted to 10μg/mL in 10× Octet kinetics buffer (Sartorius) and loaded onto hydrated Streptavidin biosensors to 1 nm shift, equilibrated in 10× Octet kinetics buffer for 150 seconds, and dipped into ACE2 ectodomains at the indicated concentrations for 300s to observe association. Dissociation was followed by dipping biosensors in a 10× Octet kinetics buffer for 300s. For dimeric ACE2 binding assays, baseline subtraction was done by subtracting the response from unloaded SA tips dipping into the highest concentration of ACE2 ectodomain used in the assays. In addition, association phases were aligned to 0 seconds and 0 response in Octet Data Analysis HT software, and the processed results were exported. For MOW15–22 binding assays, global fitting using a 1:1 binding model was carried out using the Octet Data Analysis HT software. For PnNL2018B binding assays, apparent dissociation constants were determined by plotting the average responses from 290s to 295s versus the concentration of ACE2 and fitting and one site-specific binding nonlinear fit (steady-state fitting) in GraphPad Prism 10. Sensorgrams were plotted in GraphPad Prism10.

#### Cell-cell fusion assays

A cell-cell fusion assay based on dual-split proteins (DSPs) was conducted in Caco2 cells stably expressing ACE2 receptors. To assess the S glycoprotein-receptor interaction-mediated membrane fusion between cells, group A cells were transfected with S glycoprotein and rLucN(1–155)-sfGFP1–7(1–157) expressing plasmids, while group B cells were transfected with S glycoprotein and sfGFP8–11(158–231)-rLuc(156–311) expressing plasmids. After 12 hours of transfection, both groups of cells were trypsinized, mixed, and seeded into a 96-well plate at 8 × 10^4^ cells per well. Subsequently, the cells were washed once with DMEM and then incubated with DMEM with or without indicated concentrations of TPCK-treated trypsin (Sigma-Aldrich, T8802) for 10 min at room temperature at 24 h post-transfection. Then, the cells were washed with DMEM and replenished with DMEM supplemented with 10% FBS to neutralize trypsin. Six hours later, nuclei were stained with Hoechst 33342 (1:5,000 dilution in HBSS) for 30 min at 37 °C, and images of syncytia formation with green fluorescence were subsequently captured using a fluorescence microscope (MI52-N; Mshot). To measure live-cell luciferase activity after cell-cell fusion, 20 μM of EnduRen live-cell substrate (Promega, E6481) was added to the cells in DMEM and incubated for at least 1 hour before detection using the Varioskan LUX Multi-well Luminometer (Thermo Fisher Scientific).

#### Pseudovirus production

VSV-dG-based pseudovirus (PSV) carrying trans-complementary S glycoproteins from various coronaviruses were produced following a modified protocol as previously described^73^. Briefly, HEK293T cells were transfected with plasmids expressing coronaviruses S glycoproteins. At 24 hours post-transfection, cells were transduced with 1.5×10^6^ TCID_50_ VSV-G glycoprotein-deficient VSV expressing GFP and firefly luciferase (VSV-dG-fLuc-GFP, constructed and produced in-house) diluted in DMEM with 8 μg/mL polybrene for 4–6 hours at 37 °C. After three PBS washes, the culture medium was replenished with either DMEM+10% FBS or SMM 293-TII Expression Medium (Sino Biological, M293TII), along with the presence of the neutralizing antibody (from I1-mouse hybridoma) targeting the VSV-G to eliminate the background due to any remaining VSV-dG-fLuc-GFP. Twenty-four hours later, the pseudovirus containing supernatant was clarified through centrifugation at 12,000 rpm for 5 minutes at 4°C, aliquoted, and stored at −80°C. The TCID_50_ of the PSV was calculated using the Reed-Muench method^74,75^. Heatmap presentation in Figure 3A, 3B, and S3A was set based on RLU with thresholds set based on entry background in cells without ACE2 expression. The value of P.nat ACE2 was set as a baseline of 100%, and the color of the largest value was set based on the ortholog showing the highest RLU, unless otherwise specified.

#### Single-round pseudovirus entry assay

Single-round pseudovirus entry assays were conducted using cells transiently or stably expressing different ACE2 orthologs. Approximately 3×10^4^ trypsinized cells were incubated with pseudovirus (2×10^5^ TCID_50_/100 μL) in a 96-well plate to facilitate attachment and viral entry simultaneously. Before inoculation, pseudoviruses were typically treated with 100 μg/mL TPCK-trypsin (Sigma-Aldrich, T8802). Specifically, pseudoviruses produced in serum-free SMM 293-TII Expression Medium were incubated with TPCK-treated trypsin for 10 minutes at room temperature, and the proteolytic activity was neutralized by FBS in the culture medium. Intracellular luciferase activity (Relative light units, RLU) was measured using the Bright-Glo Luciferase Assay Kit (Promega, E2620) and detected with a GloMax 20/20 Luminometer (Promega) or Varioskan LUX Multi-well Luminometer (Thermo Fisher Scientific) at 18 hours post-infection.

#### pcVSV-S amplification assay

The manipulations of propagation-competent VSV-S (pcVSV-S) were authorized by the Biosafety Committee of the State Key Laboratory of Virology, Wuhan University and conducted under BSL2 conditions. Plasmids for rescuing pcVSV-S expressing MOW15–22 and PnNL2018B S glycoproteins (pVSV-dG-GFP-MOW15–22-S and pVSV-dG-GFP-PnNL2018B-S) were generated by replacing the fLuc coding sequences with coronavirus spike coding sequences based on the vector pVSV-dG-fLuc-GFP. The propagation-competent recombinant VSVs were created by replacing the VSV-G gene with genomically encoded S glycoprotein genes and additionally incorporating a GFP-expressing cassette for visualization. Reverse genetics was applied to rescue pcVSV pseudotypes expressing MOW15–22 and PnNL2018B S glycoproteins along with a GFP reporter, following a modified protocol from previous descriptions^73^. Specifically, BHK21 cells, seeded in a 6-well plate at 80% confluence, were inoculated with 5 MOI of recombinant vaccinia virus expressing T7 RNA polymerase (vvT7, a kind gift from Mingzhou Chen’s lab, Wuhan University) for 45 minutes at 37°C. After removing vvT7, cells were subsequently transfected with pVSV-dG-GFP-S vector plasmids and helper plasmids (pVSV-dG-GFP-S: pBS-N: pBS-P: pBS-G: pBS-L=5:3:5:8:1). pcVSV-S containing supernatant (P0) was collected 48 hours post-transfection and 0.22-μm filtered to remove vvT7. For enhanced virus amplification, the passage 1 (P1) virus carrying VSV-G proteins was produced by inoculating P0 supernatant with Caco2 cells 24 hours post-transfection of plasmids expressing VSV-G protein. Subsequently, P1 viruses were further amplified in Caco2 cells stably expressing P.dav ACE2 or L.bor ACE2, without the ectopic expression of VSV-G and in the presence of anti-VSVG (I1-Hybridoma supernatant), producing passage 2 (P2) viruses carrying MOW15–22 and PnNL2018B S glycoproteins, respectively. In a typical virus amplification assay, 3×10^4^ trypsinized Caco2 cells stably expressing the indicated ACE2 were incubated with pcVSV-S (1×10^4^ TCID_50_/100 μL) in a 96-well plate in DMEM supplemented with 2% FBS with or without the indicated concations of TPCK-treated trypsin treatment. The cell nuclei were stained with Hoechst 33342 (1:10,000 dilution in HBSS) for 30 minutes at 37°C. Fluorescence images or RFU were collected at indicated time points post-infection by a fluorescence microscope (MI52-N) or a Varioskan LUX Multi-well Luminometer (Thermo Fisher Scientific).

#### Neutralization assays

For soluble ACE2 neutralization assays, serial dilutions of recombinant proteins were prepared in DMEM. VSV-based pseudoviruses (1 × 10^5^ TCID_50_ per well) were mixed with 25 μl of each dilution for 1 hour at 37 °C, and then incubated P.nat ACE2-expressing Caco2 cells seeded at 2 × 10^4^ cells per well in a 96-well plate. After 16–20 hours post-infection, the luciferase activity was measured as described in the single-round pseudotype virus entry assays. For S2P6, 76E1 antibody neutralization assays, the single-round pseudoviruses or recombinant pcVSV-S that are not pretreated by trypsin were incubated with 3-fold serial dilutions of antibodies in DMEM for 1 hour at 25°C, mixed with suspended Caco2-hACE2–3M cells in DMEM with a final concentration of 2% FBS in 96-well, followed by TPCK-treated trypsin treatment (20 μg/ml, 2 % FBS) at 4 hours post-infection without medium change. For the h11B11 neutralization assay, Caco2-hACE2–3M cells were seeded on the 96-well plate one day before infection. 3-fold serial dilutions of h11B11 were diluted by culture medium and then incubated with the cells for 1 hour at 37 °C. Pseudoviruses were then added to the cells with the presence of previously added h11B11. Neutralizing efficiencies were assessed at 18–24 hours post-infection by detecting the intracellular luciferase activity (RLU) or GFP intensity.

#### Western blot

For detecting the cellular expression of ACE2 or DPP4 receptors with C-terminal FLAG tags, cells at 24 hours post-transfection were lysed in 1% TritonX/PBS+1 mM PMSF (Beyotime, ST506) for 10 minutes at 4°C. The lysate was clarified after centrifugation at 12,000 rpm for 5 minutes at 4°C and then incubated at 98°C for 10 minutes after mixing with the 1/5 volume of 5×SDS loading buffer. Following gel electrophoresis and membrane transfer, the membranes were blocked with 5% skimmed milk in PBST for 2 hours at room temperature. Subsequently, the membrane was incubated with 1 μg/mL anti-FLAG mAb (Sigma, F1804) or anti-β-tubulin (ImmmunoWay, YM3030) mAb diluted in PBST containing 1% milk overnight at 4°C. After four washes with PBST, the blots were incubated with Horseradish peroxidase (HRP)-conjugated secondary antibody AffiniPure Goat Anti-Mouse in 1% skim milk diluted in PBST and incubated for one hour at room temperature. Finally, the blots were washed four times again by PBST and visualized using an Omni-ECL Femto Light Chemiluminescence Kit (EpiZyme, SQ201) through a ChemiDoc MP Imaging System (Bio-Rad). To examine the S glycoprotein packaging efficiency, the PSV-containing supernatant was concentrated using a 30% sucrose cushion (30% sucrose, 15 mM Tris-HCl, 100 mM NaCl, 0.5 mM EDTA) at 20,000×g for 1 hour at 4°C. The concentrated virus pellet was re-suspended in 1×SDS loading buffer and incubated at 95°C for 30 minutes, followed by western blot detecting the S glycoproteins by C-terminal HA tags and with the VSV-M serving as a loading control. Original western blot images have been deposited at Mendeley with DOI listed in the key resources table.

#### Immunofluorescence assay

Immunofluorescence assays were conducted to determine the expression levels of ACE2 orthologs with C-terminal fused FLAG tags. Specifically, the transfected cells were fixed and permeabilized by incubation with 100% methanol for 10 minutes at room temperature. Subsequently, the cells were incubated with a mouse antibody M2 (Sigma-Aldrich, F1804) diluted in PBS/1% BSA for one hour at 37°C. After one HBSS wash, the cells were incubated with Alexa Fluor 594-conjugated goat anti-mouse IgG (Thermo Fisher Scientific, A32742) secondary antibody diluted in 1% BSA/PBS for one hour at 37°C. The images were captured and merged with a fluorescence microscope (Mshot, MI52-N) after the nucleus was stained blue with Hoechst 33342 reagent (1:5,000 dilution in HBSS).

#### Recombinant glycoprotein production for cryo-EM analysis and Biolayer Interferometry

The P.dav ACE2, P.nat ACE2, and P.nat.M2 ACE2 ectodomains along with PnNL2018B and MOW15–22 RBDs were produced and purified using Expi293F. Expi293F cells were grown to a density of 3 × 10^6^ cells/mL and transfected using the ExpiFectamine 293 Transfection Kit (ThermoFisher Scientific) and expression was carried out for 4 days post-transfection at 37°C with 8% CO_2_. The proteins were purified from clarified supernatants using HisTrap HP affinity columns or Ni excel resin (Cytiva) and washed with 10–20 column volumes of 10 mM imidazole, 25 mM sodium phosphate pH 8.0, and 300 mM NaCl or 20mM imidazole, 100 mM Tris pH 8.0, and 300mM NaCl before elution with 2–10 column volumes of 300 mM imidazole, 25 mM sodium phosphate pH 8.0, and 300 mM NaCl or 300mM imidazole, 100 mM Tris pH 8.0, and 300mM NaCl. The purified proteins were then run through size exclusion chromatography using a Superdex 200 Increase 10/300 GL column (Cytiva) equilibrated in 50mM Tris pH 7.4 and 150 mM NaCl and concentrated using centrifugal filters (Amicon Ultra) before being flash frozen. For BLI, purified RBDs were biotinylated using the BirA biotin-protein ligase reaction kit (Avidity) after buffer exchanging into 50 mM Tris-HCl pH 7.4 and 150 mM NaCl using a centrifugal filter device with a MWCO of 10kDa (Amicon Ultra). Affinity purification as above-described was carried out a second time to get rid of BirA and free biotin. Purified biotinylated RBDs were concentrated using a centrifugal filter device with a MWCO of 10kDa (Amicon Ultra) and run on a Superdex200 increase 10/300 size-exclusion column (Cytiva) equilibrated in 50mM Tris pH 7.4 and 150 mM NaCl and fractions containing monomeric RBD were flash frozen and stored at −80°C until use.

P.nat.M2 ACE2 dimer with twin-strep tag was purified with Strep-Tactin XT 4Flow high-capacity resin (IBA, 2–5030-002), washed with ten column volumes of wash buffer (100 mM Tris/HCl, pH 8.0, 150 mM NaCl), and then eluted with a buffer containing 100 mM Tris/HCl, pH 8.0, 150 mM NaCl, 50 mM biotin. P.nat.M2 ACE2 and MOW15–22 RBD were initially mixed at a molar ratio of 1:1.5 and incubated for 1h on ice and then further purified in a Superose 6 Increase 10/300 GL column (Cytiva) with a buffer containing 20 Tris/HCl, pH 8.0, 150 mM NaCl. The fractions containing the complex were collected and concentrated to 1 mg/ml for further use.

#### Cryo-EM sample preparation, data collection, and data processing

Complex formation was performed by mixing a 1:2.5 molar ratio of the P.dav ACE2 ectodomain (residues 17–723 with C-terminal octa-histidine tag) with the MOW15–22 RBD (residues 351–599 with C-terminal octa-histidine tag) before incubation for 1 hour at room temperature. Cryo-EM grids of the complex were prepared using two separate methods and data were combined during data processing. For the first dataset, 3 μL of 4 mg/ml complex with 6 mM 3-[(3-Cholamidopropyl)dimethylammonio]-2-hydroxy-1-propanesulfonate (CHAPSO) were applied onto freshly glow discharged R 2/2 UltrAuFoil grids^76^ prior to plunge freezing using a vitrobot MarkIV (ThermoFisher Scientific) with a blot force of 0 and 5.5 sec blot time at 100% humidity and 22°C. 14,561 movies were collected from UltrAuFoil grids with CHAPSO detergent with a defocus range comprised between −0.2 and −3.5 μm, and stage tilt angle of 0, 20° and 30°^77^. For the second dataset, 3 μL of 0.2 mg/mL complex was added to the glow discharged side of R 2/2 UltrAuFoil grids and 1μL was added to the back side. Grids were then blotted only from the back side using a GP2 (Leica) with 6 sec blot time before plunging into liquid ethane . 8,238 movies were collected with a defocus range comprised between −0.2 and −3.5 μm. The data were acquired using an FEI Titan Krios transmission electron microscope operated at 300kV and equipped with a Gatan K3 direct detector and Gatan Quantum GIF energy filter, operated in zero-loss mode with a slit width of 20 eV. Automated data collection was carried out using SerialEM^78^ or Leginon^79^ at a nominal magnification of 105,000× with a pixel size of 0.843 Å. The dose rate was adjusted to 9 counts/pixel/s, and each movie was acquired in counting mode fractionated in 100 frames of 40 ms. Movie frame alignment, estimation of the microscope contrast-transfer function parameters, particle picking, and extraction were carried out using Warp^80^. Particles were extracted with a box size of 192 pixels with a pixel size of 1.686Å. Two rounds of reference-free 2D classification were performed using CryoSPARC^81^ to select well-defined particle images from each dataset. Particles belonging to classes with the best resolved RBD and ACE2 density were selected. To improve particle picking further, we trained the Topaz^82^ picker on Warp-picked particle sets belonging to the selected classes after 2D classification. The particles picked using Topaz were extracted and subjected to 2D classification using cryoSPARC, which improved the number of unique 2D views. The two different particle sets picked from Warp and Topaz were merged and duplicate particle picks were removed using a minimum distance cutoff of 160Å. Initial model generation was done using ab-initio reconstruction in cryoSPARC and used as references for a heterogenous 3D refinement in cryoSPARC. After two rounds of ab-initio reconstructions and heterogeneous refinements to remove junk particles, 3D refinement was carried out using non-uniform refinement with per-particle defocus refinement in cryoSPARC^83^ and the particles were transferred from cryoSPARC to Relion using pyem (https://github.com/asarnow/pyem) to be subjected to the Bayesian polishing procedure implemented in Relion^84^ during which particles were re-extracted with a box size of 320 pixels and a pixel size of 1.0 Å. After ab-initio reconstruction and heterogeneous refinement to remove without bound RBD, subsequent 3D refinement used non-uniform refinement along with per-particle defocus refinement in cryoSPARC. To further improve the density of the RBD/ACE2 interface, local refinement was performed using cryoSPARC with a soft mask comprising one ACE2 peptidase domain and the bound RBD. Local resolution estimation, filtering, and sharpening were carried out using cryoSPARC to yield the final reconstruction at 2.8 Å resolution comprising 705,956 particles. Reported resolutions are based on the 0.143 gold-standard Fourier shell correlation (FSC) criterion and Fourier shell correlation curves were corrected for the effects of soft masking by high-resolution noise substitution^85,86^.

For the P.nat.M2 ACE2 bound-PnNL2018B RBD structure, complex formation was performed by mixing a 1:1.2 molar ratio of the P.nat.M2 ACE2 ectodomain (residues 17–740) with the PnNL2018 RBD (residues 361–606) before incubation for 1 hour at room temperature. 3 μL of 5.5 mg/ml complex with 6 mM CHAPSO were applied onto freshly glow discharged R 2/2 UltrAuFoil grids prior to plunge freezing using a vitrobot MarkIV (ThermoFisher Scientific) with a blot force of 0 and 5.5 sec blot time at 100% humidity and 22°C. 8,265 movies were collected from UltrAuFoil grids with a defocus range comprised between −0.2 and −3.5 μm. The overall data processing methods were the same as that for the P.dav ACE2-bound MOW15–22 RBD structure except that non-uniform refinement used C2 symmetry. After symmetry-expansion, the final dataset contained 1,555,546 asymmetric units used for the final local refinement with a soft mask comprising one ACE2 peptidase domain and the bound RBD resulting in a 2.4 Å resolution reconstruction. We identified at most one MOW15–22 RBD bound to each P.dav ACE2 dimer whereas particles corresponding to complexes had two PnNL2018B RBDs bound to each P.nat.M2 ACE2 dimer. More details are shown in Figure S7.

For P.nat.M2 ACE2 and MOW15–22 RBD complex, 3.5 μL of the purified complex at 0.8 mg/mL was applied to glow-discharged Cu R1.2/1.3 holey carbon grid (200 mesh, Quantifoil) with a final concentration of 1% glycerol. After incubation for 20 s, the grids were blotted with force 0 for 2 s at 4°C and 100% humidity, and plunge-frozen into liquid ethane using Vitrobot Mark IV (FEI Thermo Fisher). Grids were then transferred to a CRYO ARM 300 electron microscope (JEOL, Japan) operating at 300 kV equipped with a K3 direct electron detector (Gatan, USA). Cryo-EM images were recorded automatically using Serial-EM software with a super-resolution pixel size of 0.475 Å /pixel at defocus values ranging from −0.5 to −2.5 μm at a calibrated magnification of 50,000. Data were collected at a frame rate of 40 frames per second. The total electron dose was 40 e−/Å2. 4,214 movies were collected and subjected to patch motion correction and patch CTF estimation in cryoSPARC. Particles were automatically picked with a diameter of 120 Å from 200 micrographs. ~50,000 particles representing good classes were used to train a topaz model for auto-picking. A subset of 785,038 particles was selected after two rounds of 2D classification, followed by an ab initio reconstitution and heterogeneous refinement. One class representing intact P.nat.M2 ACE2-MOW15–22 RBD complex was further selected for heterogeneous refinement requesting three classes. One class containing 244,733 particles with good features was subjected to non-uniform refinement, yielding a 3.3 Å reconstruction.

#### Model building and refinement

UCSF Chimera^87^ was used to rigid-body dock models into the sharpened cryo-EM map and adjustments and refinement were carried out with Coot^88^ and Rosetta^89,90^ using sharpened and unsharpened maps. Validation used Molprobity^91^, Phenix^92^ and Privateer^93^.

#### Bioinformatic and structural analysis

Sequence alignments of different bats ACE2 were performed using either the MUSCLE algorithm by MEGA-X (version 10.1.8) or ClustalW software (https://www.genome.jp/tools-bin/clustalw) with slight position adjustment to align indels. Phylogenetic trees were generated using the maximal likelihood method in IQ-TREE (http://igtree.cibiv.univie.ac.at/) (1000 Bootstraps) and refined with iTOL (v6) (https://itol.embl.de/). The structures of PnNL2018B, and HKU31 RBDs were predicted using alphaFold2.ipynb-Colaboratory^26^. The structure of NeoCoV RBD and P.pip ACE2 complex (7WPO), MERS-CoV RBD (4KQZ), HKU4 RBD (4QZV), HKU5 RBD (5XGR), MOW15–22 RBD, HKU31 RBD, and PnNL2018B RBD were visualized and analyzed using the ChimeraX (V.1.7.1). Analysis of buried surface area and identification of interface residues were assisted by PISA^70^.

### QUANTIFICATION AND STATISTICAL ANALYSIS

Most experiments related to pseudovirus infection were conducted 2–3 times with 2–4 biological repeats, technical repeats are indicated in the legends. Representative results were shown. All data were presented by MEAN , MEAN ± SD and MEAN ± SEM . Unpaired two-tailed t-tests were conducted for all statistical analyses for two independent groups using GraphPad Prism 8. *P*<0.05 was considered significant. *: *P*<0.05, **: *P*<0.01, ***: *P*<0.005, and ****: *P*<0.001, NS: not significant. No data were excluded for data analysis.

## Supplementary Material

Fig S2 **Figure S2. Trypsin-dependence of bat ACE2-mediated MOW15–22 membrane fusion and PSV entry, related to** Figure 3. (**A-B**) Heat map (A) and bar graph (B) of MOW15–22 PSV entry efficiency mediated by several ACE2 orthologs in the presence or absence of 100 μg/mL TPCK-treated trypsin. P.dav ACE2 was set at 100% as it promotes the most effective entry (for MOW15–22). Red highlights the two bat ACE2s supporting efficient MOW15–22 RBD binding. The dashed line indicates the background signal. Data are represented as mean ± SD and unpaired two-tailed t-tests. n=3 biological replicates. (**C-D**) MOW15–22 S-mediated cell-cell membrane fusion in HEK293T cells stably expressing the indicated ACE2 orthologs in the presence of various concentrations of TPCK-treated trypsin. Fusion efficiency is indicated by GFP intensity (C) and live-cell Renilla luciferase activity (D) through the reconstitution of dual-split reporter proteins (DSPs). Data are represented as mean ± SD and unpaired two-tailed t-tests. n=3 biological replicates. (**E-F**) MOW15–22 PSV entry efficiency in HEK293T cells stably expressing ACE2 orthologs with the indicated concentration of TPCK-treated trypsin, as indicated by GFP intensity (E) and luciferase (F). n=3 biological replicates for F. Scale bars in C and E:200 μm.

Fig S3 **Figure S3. ACE2 determinants critical for NeoCoV/PDF-2180 do not impact MOW15–22/PnNL2018B ACE2 recognition, related to** Figure 4. (**A**) Structural presentation of the four host range determinants (A-D) critical for P.Pip ACE2 recognition by NeoCoV (PDB 7WPO). (**B**) Schematic illustration of P.dav and P.par ACE2 mutants with sequences of indicated determinants replaced by residues unfavorable for NeoCoV recognition. Glycosylation sites in determinants A and C are indicated with ￥. (**C**) MOW15–22 and NeoCoV RBD binding to HEK293T cells transiently expressing the indicated wild-type (WT) or mutants ACE2 orthologs. The expression level of indicated ACE2 orthologs was verified by immunofluorescence (C, top panel). Scale bars: 100 μm. (**D**) Western blot analysis of the expression of indicated WT and mutated ACE2 in HEK293T cells. MW: molecular weight. (**E-F**) PSV entry efficiency of PnNL2018B in HEK293T cells transiently expressing the L.bor (E) and P.pip (F) ACE2 mutants affecting NeoCoV receptor recognition. Data are represented as mean ± SD for n=3 biological replicates. RLU: relative light unit.

Fig S1 **Figure S1. Validation of the expression of ACE2 orthologs and comparision of binding efficiency between MOW15–22 S_1_-hFc and MOW15–22 RBD-hFc, related to** Figure 3. (**A-B**) Immunofluorescence analysis of the expression of bat (A) or non-bat mammalian (B) ACE2 orthologs in HEK293T cells by detecting 3×FLAG tags fused to the C-terminal of the receptors. Scale bars:100 μm. (**C**) Immunofluorescence analysis of binding of the MOW15–22 RBD or S_1_ subunit at the indicated concentrations to HEK293T cells transiently expressing the indicated ACE2 orthologs. (**D**) Binding of hFc-fused recombinant proteins (50 nM) comprising different domains of the MOW15–22 S_1_ subunit to HEK293T cells transiently expressing the selected mammalian ACE2 orthologs supporting MOW15–22 PSV entry. PnNL2018B S_1_-hFc subunit was included as a control. Scale bars: 100

Fig S5 **Figure S5. Cryo-EM data processing workflow for the P.dav ACE2-bound MOW15–22 and P.nat.M2 ACE2-bound PnNL2018B RBD complex, related to** Figure 5. (**A-B**) Representative electron micrograph (A) and 2D class averages (B) of the P.dav ACE2-bound MOW15–22 RBD complex embedded in vitreous ice. The scale bars represent 100 nm and 200Å, respectively. (**C**) Gold-standard Fourier shell correlation curve. The 0.143 cutoff is indicated by a horizontal dashed line. (**D**) Local resolution estimation was calculated using cryoSPARC and plotted on the sharpened map. The angular distribution calculated in cryoSPARC for particle projections is also shown as a heat map. (**E**) Data processing flowchart. CTF: contrast transfer function; NUR: non-uniform refinement. (**F-G**) Representative electron micrograph (F) and 2D class averages (G) of the P.nat M2 ACE2-bound PnNL2018B RBD complex embedded in vitreous ice. The scale bars represent 100 nm and 100Å, respectively. (**H**) Gold-standard Fourier shell correlation curve. The 0.143 cutoff is indicated by a horizontal dashed line. (**I**) Local resolution estimation was calculated using cryoSPARC and plotted on the sharpened map. The angular distribution calculated in cryoSPARC for particle projections is also shown as a heat map. (**J**) Data processing flowchart. CTF: contrast transfer function; NUR: non-uniform refinement.

Fig S4 **Figure S4. Mapping of ACE2 host range determinants for MOW15–22 and PnNL2018B, related to** Figure 4. (**A**) Schematic strategy of sequence swaps between M.bla and P.dav ACE2s. (**B**) MOW15–22 PSV entry into HEK293T cells transiently expressing the indicated ACE2 chimeras. The swaps between residues 400–450 that enable the phenotype switch are highlighted in red. (**C-D**) Determinant mapping based on sequence swaps between residues 400–500 of M.bla and P.dav ACE2. Schematic illustration of chimeric ACE2 mutants enhancing M.bla ACE2 binding (C) or abolishing P.dav ACE2 binding (D). RBD binding to (middle) and pseudovirus entry into (right) HEK293T cells transiently expressing the indicated ACE2 swap mutants are shown. The critical Y430_P.davACE2_ residue abolishing the corresponding N-glycosylation site in M.bla is highlighted in red. (**E-F**) Binding efficiencies of recombinant proteins comprising different domains of the MOW15–22 (E) or PnNL2018B (F) S_1_ subunit to Caco2 cells stably expressing P.nat ACE2 or P.nat ACE2-C432N. (**G-H**) Immunofluorescence analyses of PnNL2018B RBD and S_1_ subunit binding to HEK293T cells transiently expressing ACE2 chimeras with sequence swaps between P.pip ACE2-N432C and P.nat ACE2 (G) or P.pip ACE2–3M (N432C/E589K/K597E) mutant and P.nat ACE2 (H). Data are represented as mean ± SD for n=3 biological replicates in B-D. Scale bars: 100 μm.

Fig S6 **Figure S6. Validation of critical residues for interactions between MOW15–22/PnNL2018B and different ACE2 orthologs, related to** Figure 5. (**A**) P. nat ACE2 mutants with reduced PnNL2018B RBD or S_1_ binding due to unfavorable substitutions of key interacting residues. (**B**) P.pip, human, and P.kuh ACE2 mutants with enhanced ability to promote PnNL2018B RBD or S_1_ binding. (**C-D**) Marmoset (C.jac) ACE2 mutants promoting enhanced MOW15–22 and PnNL2018B RBD or S_1_ binding and PSV entry or abolishing receptor function via introducing the N432 glycan knock-in mutation. MEAN ± SD and unpaired two-tailed t-tests. n=3 biological replicates for D. (**E-F**) MOW15–22 RBM mutants with reduced ability to utilize P.dav and P. nat ACE2 orthologs for RBD binding (E) and pseudovirus entry (F). Data are represented as mean ± SD and unpaired two-tailed t-tests for F. n=3 biological replicates. (**G**) VSV packaging efficiencies of MOW15–22 S harboring the indicated mutations.VSV-M was used as a loading control. Scale bars in A, B, C, and E :100 μm.

Fig S7 **Figure S7. Evaluation of entry inhibitors against PnNL2018B and MOW15–22 pseudovirus entry, related to** Figure 6 and Figure 7. (**A-C**) BLI analyses of binding kinetics of soluble dimeric ACE2 ectodomains from wildtype (WT) P.nat ACE2 (**A**), P.nat.M1 ACE2 (**B**), or P.nat.M2 ACE2 (**C**) to the immobilized PnNL2018B S_1_-hFc. Analysis was conducted with global fitting (1:1 binding model) and the fit to the data is shown in black. (**D-E**) Representative electron micrograph and 2D class averages of the P.nat.M2 ACE2 bound MOW15–22 RBD complex embedded in vitreous ice. (**F**) Flowchart of cryo-EM data processing. (**G**) Fourier shell correlation (FSC) curve was calculated using two independent half maps, and resolution was estimated using the FSC=0.143 cutoff. (**H**) Neutralization efficiency of MERS-CoV RBD-directed nanobodies against MERS-CoV and MOW15–22 pseudotyped viruses. Data are represented as mean ± SD and unpaired two-tailed t-tests. n=3 biological replicates. (**I-J**) Sequence alignment displaying corresponding sequences of S2P6 (I) and 76E1 (J) epitopes of indicated coronaviruses. Red dashed box: S2P6 epitope. The MOW15–22 residue numbering is shown in B and C. (**K**) Dose-dependent inhibition of NeoCoV pseudovirus entry by antibodies targeting the stem helix (S2P6) or the S_2_’/fusion peptide (76E1) in HEK293T cells expressing P.pip ACE2. (**L**) The hACE2-specific antibody h11B11 does not inhibit MERS-CoV pseudovirus entry in Caco2-hACE2–3M cells. n=2 biological replicates for D and E. (**M**) Dose-dependent inhibition of pcVSV-MOW15–22-S propagation by h11B11. BSA was used as a negative control. Scale bars: 200

Table S1

Table S2

Table S3

## Figures and Tables

**Table:** 

REAGENT or RESOURCE	SOURCE	IDENTIFIER
Antibodies		
Alexa Fluor 488-conjugated goat anti-human IgG	Thermo Fisher Scientific	Cat#A11013; RRID:AB_2534080
Mouse anti-FLAG antibody M2	Sigma-Aldrich	Cat#F1804; RRID:AB_262044
Anti-VSV-M(23H12)Antibody	kerafast	Cat# EB0011; RRID:AB_2734773
Anti-HA-tag mAb	MBL	Cat# M180-3; RRID:AB_10951811
Alexa Fluor 594-conjugated goat anti-mouse IgG	Thermo Fisher Scientific	Cat#A32742; RRID:AB_2762825
Goat anti-human IgG(H+L) Cross Absorbed Secondary Antibody	Thermo Fisher Scientific	Cat#A11013; RRID:AB_2534080
β-Tubulin mAb	ImmmunoWay	Cat#YM3030; RRID:AB_3661958
h11b11	Du et Zl. ^45^	N/A
76E1	Sun et al. ^44^	N/A
S2P6	Pinto et al. ^43^	N/A
Bacterial and virus strains		
VVT7	Mingzhou Chen’s lab, Wuhan University	N/A
pcVSV-MOW15-22-S	This study	N/A
pcVSV-PnNL2018B-S	This study	N/A
VSV-dG-fLuc-GFP	Ma et al. ^24^	N/A
Chemicals, peptides, and recombinant proteins		
TPCK-treated trypsin	Sigma-Aldrich	Cat#T8802
Polybrene	Beyotime	Cat#C0351
Dulbecco’s Modified Eagle Medium	Monad, China	Cat#CC00101S
Penicillin/Streptomycin	Biosharp	Cat#BL505A
Fetal Bovine Serum	Excell	Cat#FSP500-11I323
GeneTwin reagent	Biomed	Cat#TG101
Trypsin-EDTA (0.25%)	Gibco	Cat#25200072
SMM 293-TII Expression Medium	Sino Biological	Cat#M293TII
Pierce Protein A/G Plus Agarose	Thermo Scientific	Cat#20424
1M Tris-HCI, pH 8.0	Thermo Scientific	Cat#15568025
Strep-Tactin XT 4Flow high-capacity resin	IBA	Cat#2-5030-002
Hanks’ Balanced Salt Solution	ServiceBio	Cat#G4204
Poly-L-lysine hydrobromide	Sigma	Cat#P1399
PMSF	Beyotime	Cat#ST506
Triton X-100	Solarbio	Cat#T8200
GelGreen	Biosharp	Cat#BS355A
D-Biotin	Shanghai yuanye Bio-Technology Co., Ltd	Cat#S13004
EK1C4 peptide	LuLu’s lab, Fudan University	N/A
E64d	Aladdin	Cat#E123225
Camostat	MedChemExpress	Cat#HY-13512
Bafilomycin A1	MedChemExpress	Cat#HY-100558
Critical commercial assays		
Bright-Glo Luciferase Assay Kit	Promega	Cat#E2650
EnduRen live-cell substrate	Promega	Cat#E6481
Omni-ECL Femto Light Chemiluminescence Kit	EpiZyme	Cat#SQ201
Omni-Easy Instant BCA Protein Assay Kit	Epizyme	Cat#SZJ102
Genejet gel extraction kit	Thermo	Cat#K0692
Deposited data		
P.nat.M2 ACE2-bound MOW15-22 RBD cryo-EM structure	This study	EMD 60483, PDB:8ZUF
P.dav ACE2-bound MOW15-22 RBD cryo-EM structure	This study	EMD 45253, PDB: 9C6O
P.nat.M2 ACE2-bound PnNL2018B RBD cryo-EM structure	This study	EMD 46691, PDB: 9DAK
P.nat ACE2 and P.nat DPP4 coding sequences	This study	https://data.mendeley.com/preview/mw3gyj7ncr?a=6a5b0b4f-ad01-4f5c-b573-f73a89404b1a
Original western blot images	This study	https://data.mendeley.com/preview/wjns8j3z6y?a=3d8afefb-cd8b-4bba-aeab-3948f7d94236
Experimental models: Cell lines		
HEK293T	ATCC	CRL-3216
HEK293T	ATCC	CRL-11268
Caco2	ATCC	HTB-37
BHK21	ATCC	CCL-10
I1-Hybridoma cell lines	ATCC	CRL-2700
Expi293F	ATCC	A14527
Recombinant DNA		
pVSV-dG-GFP-MOW15-22-S	This paper	N/A
pVSV-dG-GFP-PnNL2018B-S	This paper	N/A
rLucN(1-155)-sfGFP1-7(1-157)	This paper	N/A
sfGFP8-11(158-231)-rLuc(156-311)	This paper	N/A
Software and algorithms		
MEGA-X version 10.1.8	Kumar et al. ^65^	https://www.megasoftware.net/
IQ-TREE	Minh et al. ^66^	http://igtree.cibiv.univie.ac.at/
iTOL (v6)	Letunic and Bork ^67^	https://itol.embl.de/
UCSF ChimeraX (V.1.7.1)	Goddard et al. ^68^	https://www.rbvi.ucsf.edu/chimerax
ColabFold v1.5.5: AlphaFold2 using MMseqs2	Mirdita et al. ^69^	https://colab.research.google.com/github/sokrypton/ColabFold/blob/main/AlphaFold2.ipynb
PDBePISA	Krissinel and Henrick ^70^	https://www.ebi.ac.uk/pdbe/pisa/pistart.html
ClustalW	Thompson et al. ^71^	https://www.genome.jp/tools-bin/clustalw
Other		
MERS-CoV RBD structure	Lu et al. ^17^	PDB:4KQZ
HKU4 RBD structure	Wang et al. ^13^	PDB:4QZV
NeoCoV RBD structure	Xiong et al. ^9^	PDB:7WPO
HKU5 RBD structure	Han et al. ^72^	PDB:5XGR
