# Supplementary figures and images for: Multiple independent acquisitions of ACE2 usage in MERS-related coronaviruses
Source: Cell. Author manuscript; Available in PMC 2025 Aug 18. (PMC12360793; doi:10.1016/j.cell.2024.12.031)

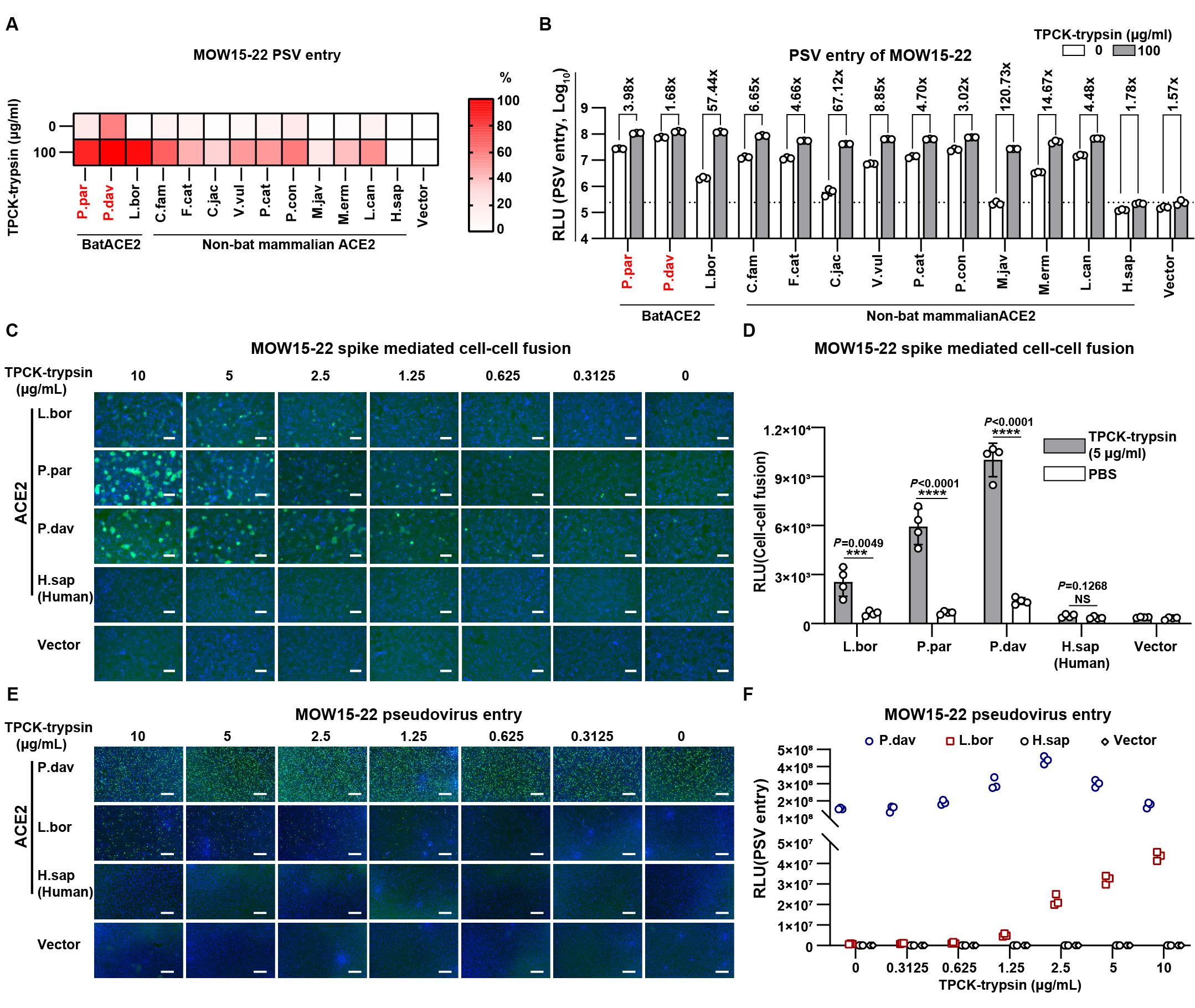

Supplement: Fig S2 — Figure S2. Trypsin-dependence of bat ACE2-mediated MOW15–22 membrane fusion and PSV entry, related to Figure 3. (A-B) Heat map (A) and bar graph (B) of MOW15–22 PSV entry efficiency mediated by several ACE2 orthologs in the presence or absence of 100 μg/mL TPCK-treated trypsin. P.dav ACE2 was set at 100% as it promotes the most effective entry (for MOW15–22). Red highlights the two bat ACE2s supporting efficient MOW15–22 RBD binding. The dashed line indicates the background signal. Data are represented as mean ± SD and unpaired two-tailed t-tests. n=3 biological replicates. (C-D) MOW15–22 S-mediated cell-cell membrane fusion in HEK293T cells stably expressing the indicated ACE2 orthologs in the presence of various concentrations of TPCK-treated trypsin. Fusion efficiency is indicated by GFP intensity (C) and live-cell Renilla luciferase activity (D) through the reconstitution of dual-split reporter proteins (DSPs). Data are represented as mean ± SD and unpaired two-tailed t-tests. n=3 biological replicates. (E-F) MOW15–22 PSV entry efficiency in HEK293T cells stably expressing ACE2 orthologs with the indicated concentration of TPCK-treated trypsin, as indicated by GFP intensity (E) and luciferase (F). n=3 biological replicates for F. Scale bars in C and E:200 μm. [file NIHMS2101199-supplement-Fig_S2.jpg]

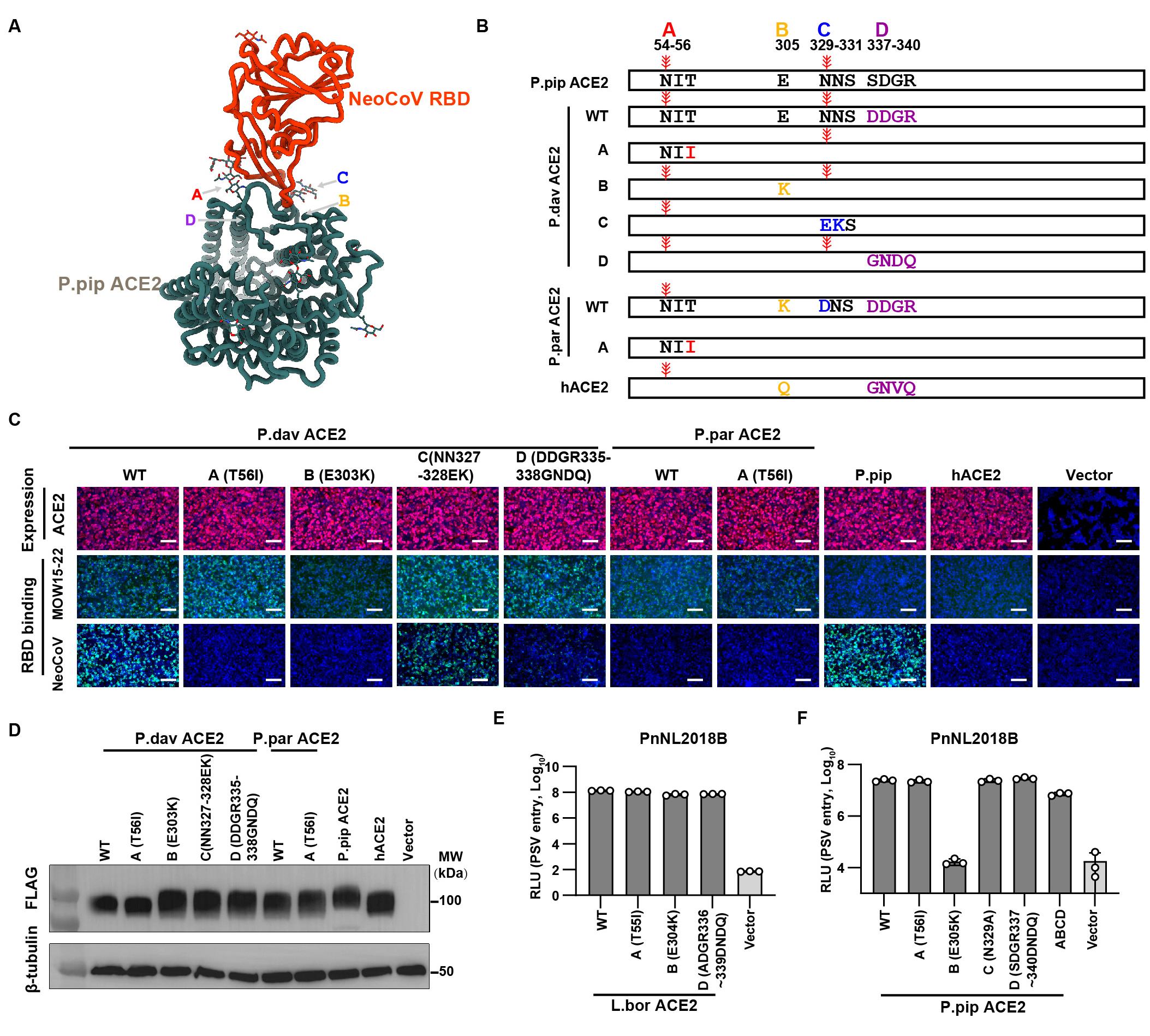

Supplement: Fig S3 — Figure S3. ACE2 determinants critical for NeoCoV/PDF-2180 do not impact MOW15–22/PnNL2018B ACE2 recognition, related to Figure 4. (A) Structural presentation of the four host range determinants (A-D) critical for P.Pip ACE2 recognition by NeoCoV (PDB 7WPO). (B) Schematic illustration of P.dav and P.par ACE2 mutants with sequences of indicated determinants replaced by residues unfavorable for NeoCoV recognition. Glycosylation sites in determinants A and C are indicated with ￥. (C) MOW15–22 and NeoCoV RBD binding to HEK293T cells transiently expressing the indicated wild-type (WT) or mutants ACE2 orthologs. The expression level of indicated ACE2 orthologs was verified by immunofluorescence (C, top panel). Scale bars: 100 μm. (D) Western blot analysis of the expression of indicated WT and mutated ACE2 in HEK293T cells. MW: molecular weight. (E-F) PSV entry efficiency of PnNL2018B in HEK293T cells transiently expressing the L.bor (E) and P.pip (F) ACE2 mutants affecting NeoCoV receptor recognition. Data are represented as mean ± SD for n=3 biological replicates. RLU: relative light unit. [file NIHMS2101199-supplement-Fig_S3.jpg]

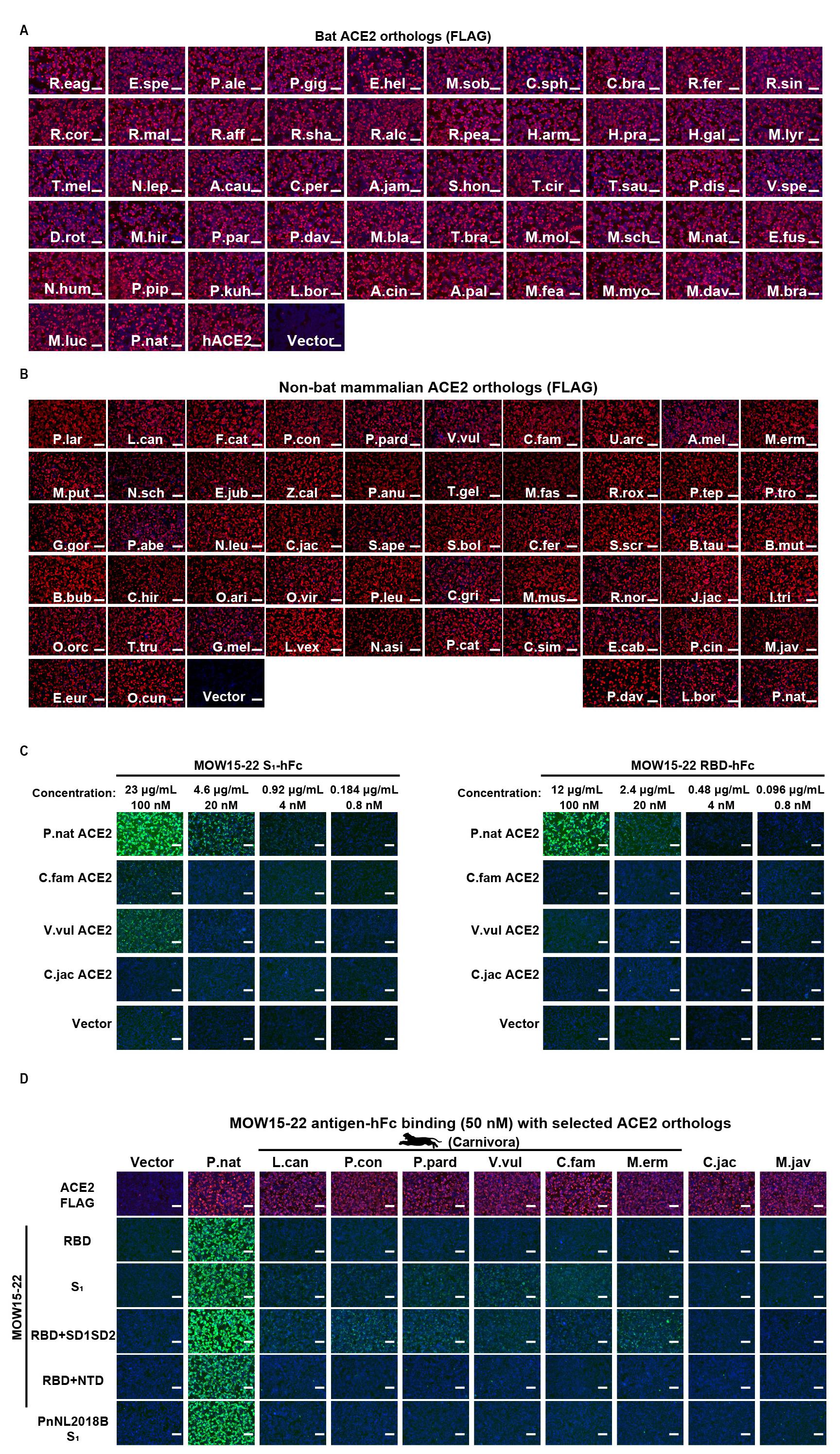

Supplement: Fig S1 — Figure S1. Validation of the expression of ACE2 orthologs and comparision of binding efficiency between MOW15–22 S1-hFc and MOW15–22 RBD-hFc, related to Figure 3. (A-B) Immunofluorescence analysis of the expression of bat (A) or non-bat mammalian (B) ACE2 orthologs in HEK293T cells by detecting 3×FLAG tags fused to the C-terminal of the receptors. Scale bars:100 μm. (C) Immunofluorescence analysis of binding of the MOW15–22 RBD or S1 subunit at the indicated concentrations to HEK293T cells transiently expressing the indicated ACE2 orthologs. (D) Binding of hFc-fused recombinant proteins (50 nM) comprising different domains of the MOW15–22 S1 subunit to HEK293T cells transiently expressing the selected mammalian ACE2 orthologs supporting MOW15–22 PSV entry. PnNL2018B S1-hFc subunit was included as a control. Scale bars: 100 [file NIHMS2101199-supplement-Fig_S1.jpg]

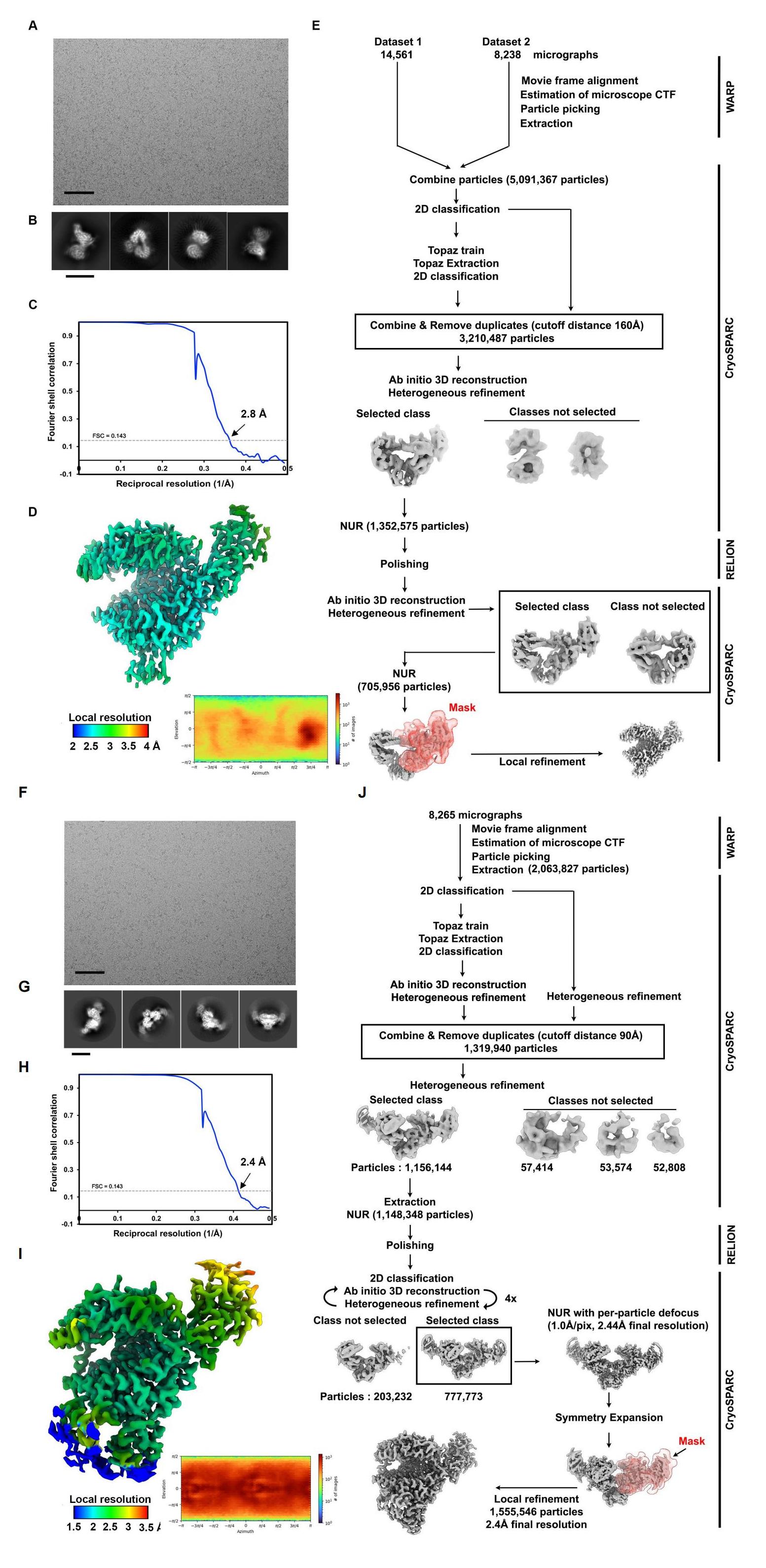

Supplement: Fig S5 — Figure S5. Cryo-EM data processing workflow for the P.dav ACE2-bound MOW15–22 and P.nat.M2 ACE2-bound PnNL2018B RBD complex, related to Figure 5. (A-B) Representative electron micrograph (A) and 2D class averages (B) of the P.dav ACE2-bound MOW15–22 RBD complex embedded in vitreous ice. The scale bars represent 100 nm and 200Å, respectively. (C) Gold-standard Fourier shell correlation curve. The 0.143 cutoff is indicated by a horizontal dashed line. (D) Local resolution estimation was calculated using cryoSPARC and plotted on the sharpened map. The angular distribution calculated in cryoSPARC for particle projections is also shown as a heat map. (E) Data processing flowchart. CTF: contrast transfer function; NUR: non-uniform refinement. (F-G) Representative electron micrograph (F) and 2D class averages (G) of the P.nat M2 ACE2-bound PnNL2018B RBD complex embedded in vitreous ice. The scale bars represent 100 nm and 100Å, respectively. (H) Gold-standard Fourier shell correlation curve. The 0.143 cutoff is indicated by a horizontal dashed line. (I) Local resolution estimation was calculated using cryoSPARC and plotted on the sharpened map. The angular distribution calculated in cryoSPARC for particle projections is also shown as a heat map. (J) Data processing flowchart. CTF: contrast transfer function; NUR: non-uniform refinement. [file NIHMS2101199-supplement-Fig_S5.jpg]

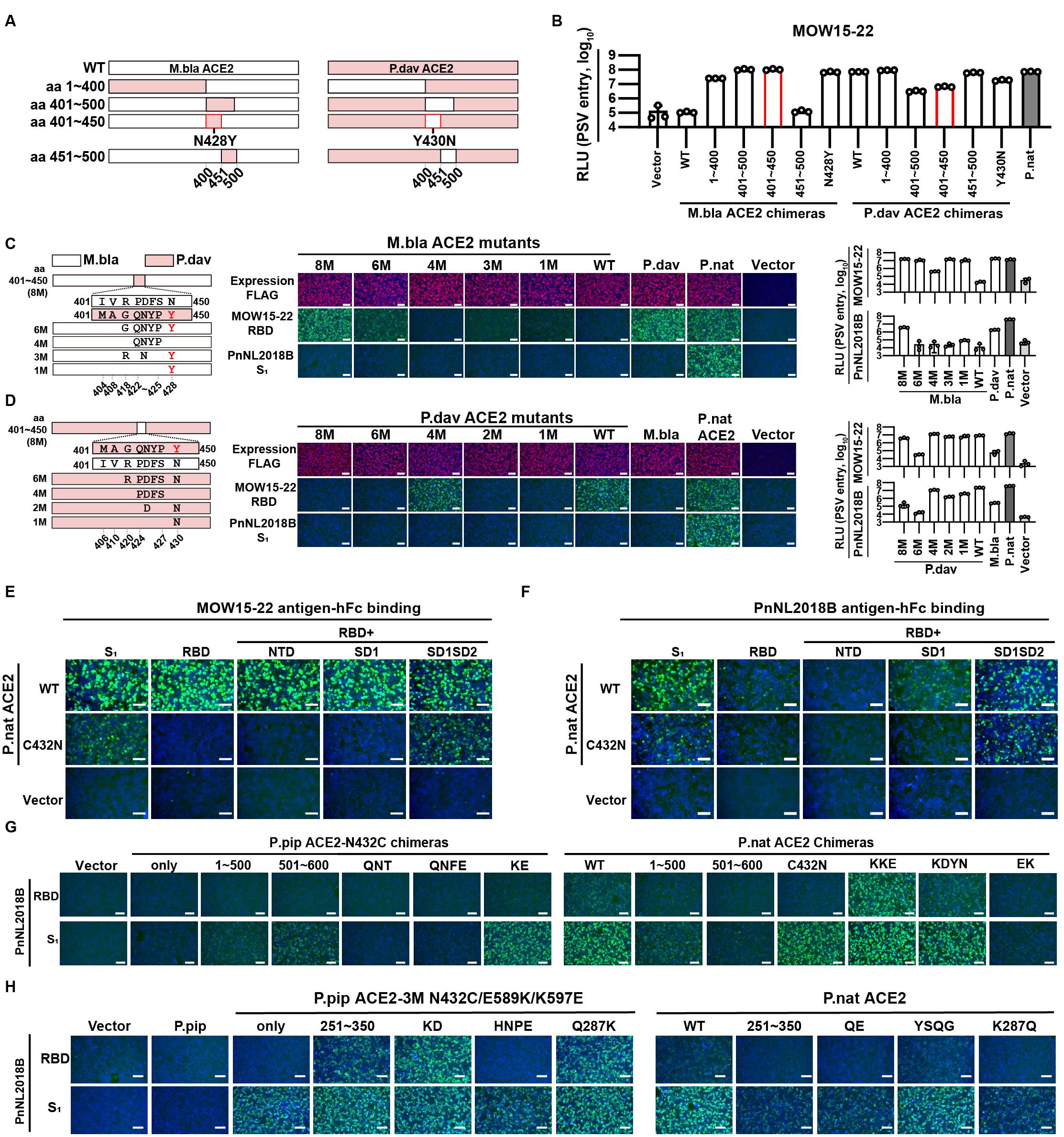

Supplement: Fig S4 — Figure S4. Mapping of ACE2 host range determinants for MOW15–22 and PnNL2018B, related to Figure 4. (A) Schematic strategy of sequence swaps between M.bla and P.dav ACE2s. (B) MOW15–22 PSV entry into HEK293T cells transiently expressing the indicated ACE2 chimeras. The swaps between residues 400–450 that enable the phenotype switch are highlighted in red. (C-D) Determinant mapping based on sequence swaps between residues 400–500 of M.bla and P.dav ACE2. Schematic illustration of chimeric ACE2 mutants enhancing M.bla ACE2 binding (C) or abolishing P.dav ACE2 binding (D). RBD binding to (middle) and pseudovirus entry into (right) HEK293T cells transiently expressing the indicated ACE2 swap mutants are shown. The critical Y430P.davACE2 residue abolishing the corresponding N-glycosylation site in M.bla is highlighted in red. (E-F) Binding efficiencies of recombinant proteins comprising different domains of the MOW15–22 (E) or PnNL2018B (F) S1 subunit to Caco2 cells stably expressing P.nat ACE2 or P.nat ACE2-C432N. (G-H) Immunofluorescence analyses of PnNL2018B RBD and S1 subunit binding to HEK293T cells transiently expressing ACE2 chimeras with sequence swaps between P.pip ACE2-N432C and P.nat ACE2 (G) or P.pip ACE2–3M (N432C/E589K/K597E) mutant and P.nat ACE2 (H). Data are represented as mean ± SD for n=3 biological replicates in B-D. Scale bars: 100 μm. [file NIHMS2101199-supplement-Fig_S4.jpg]

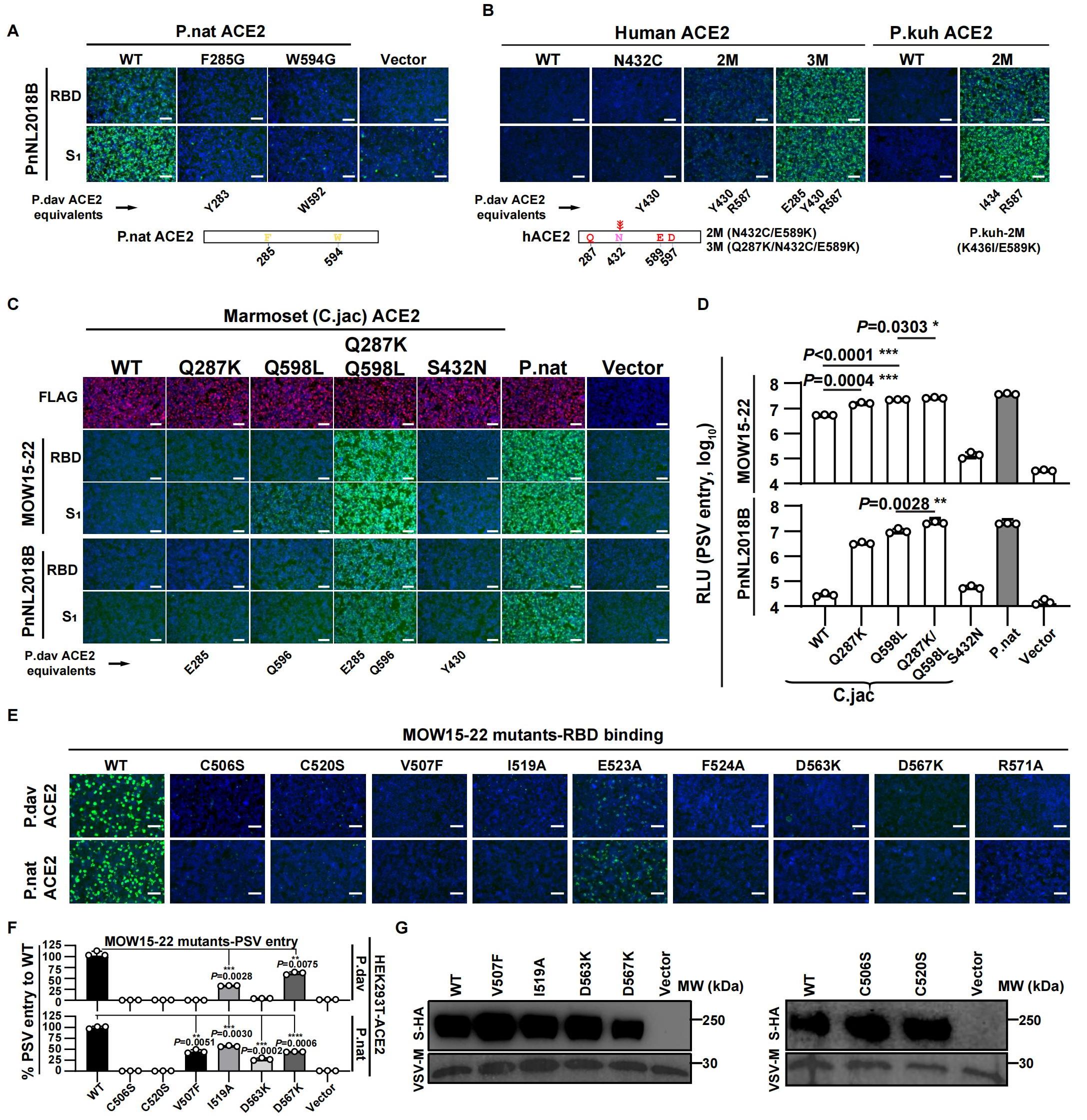

Supplement: Fig S6 — Figure S6. Validation of critical residues for interactions between MOW15–22/PnNL2018B and different ACE2 orthologs, related to Figure 5. (A) P. nat ACE2 mutants with reduced PnNL2018B RBD or S1 binding due to unfavorable substitutions of key interacting residues. (B) P.pip, human, and P.kuh ACE2 mutants with enhanced ability to promote PnNL2018B RBD or S1 binding. (C-D) Marmoset (C.jac) ACE2 mutants promoting enhanced MOW15–22 and PnNL2018B RBD or S1 binding and PSV entry or abolishing receptor function via introducing the N432 glycan knock-in mutation. MEAN ± SD and unpaired two-tailed t-tests. n=3 biological replicates for D. (E-F) MOW15–22 RBM mutants with reduced ability to utilize P.dav and P. nat ACE2 orthologs for RBD binding (E) and pseudovirus entry (F). Data are represented as mean ± SD and unpaired two-tailed t-tests for F. n=3 biological replicates. (G) VSV packaging efficiencies of MOW15–22 S harboring the indicated mutations.VSV-M was used as a loading control. Scale bars in A, B, C, and E :100 μm. [file NIHMS2101199-supplement-Fig_S6.jpg]

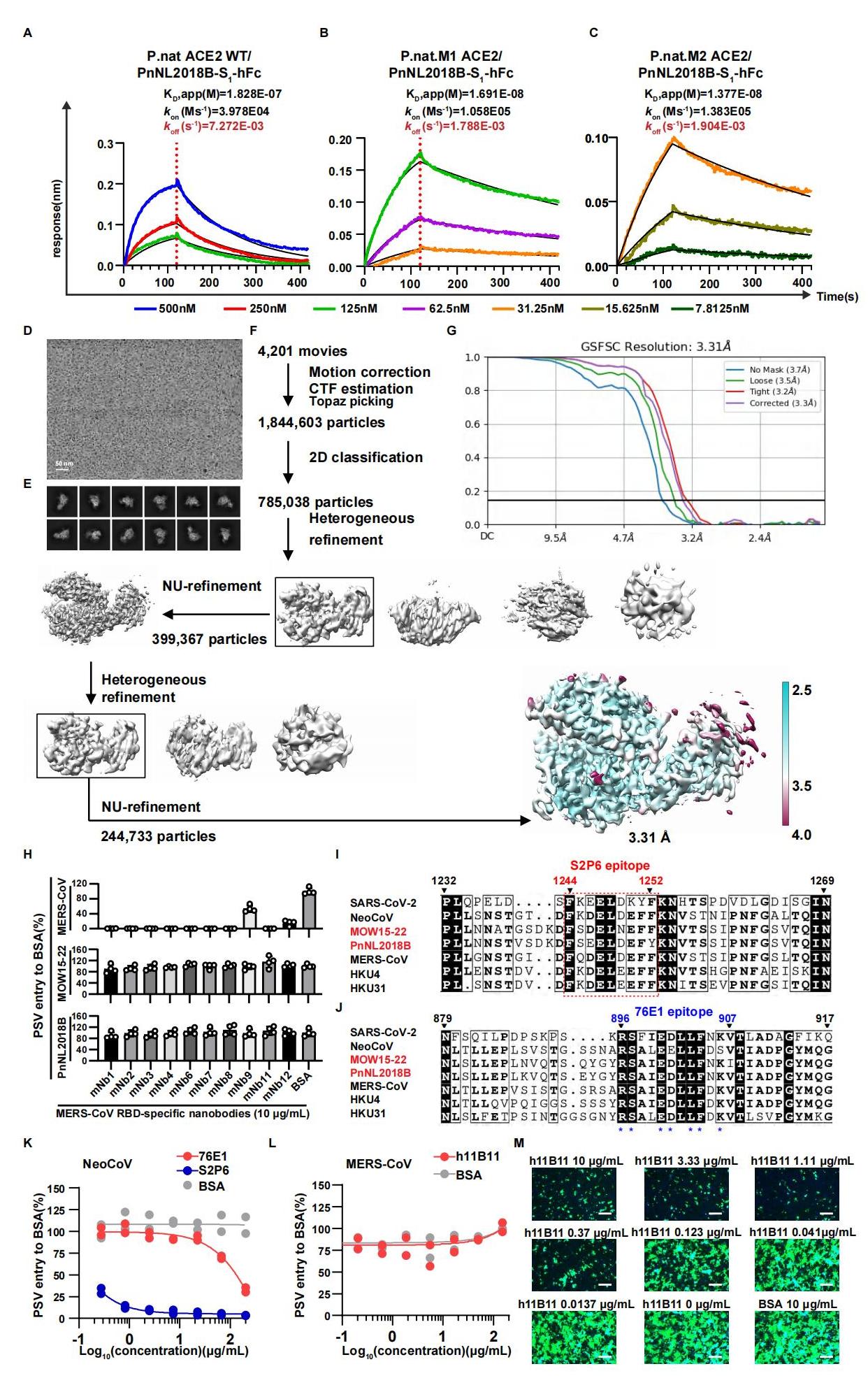

Supplement: Fig S7 — Figure S7. Evaluation of entry inhibitors against PnNL2018B and MOW15–22 pseudovirus entry, related to Figure 6 and Figure 7. (A-C) BLI analyses of binding kinetics of soluble dimeric ACE2 ectodomains from wildtype (WT) P.nat ACE2 (A), P.nat.M1 ACE2 (B), or P.nat.M2 ACE2 (C) to the immobilized PnNL2018B S1-hFc. Analysis was conducted with global fitting (1:1 binding model) and the fit to the data is shown in black. (D-E) Representative electron micrograph and 2D class averages of the P.nat.M2 ACE2 bound MOW15–22 RBD complex embedded in vitreous ice. (F) Flowchart of cryo-EM data processing. (G) Fourier shell correlation (FSC) curve was calculated using two independent half maps, and resolution was estimated using the FSC=0.143 cutoff. (H) Neutralization efficiency of MERS-CoV RBD-directed nanobodies against MERS-CoV and MOW15–22 pseudotyped viruses. Data are represented as mean ± SD and unpaired two-tailed t-tests. n=3 biological replicates. (I-J) Sequence alignment displaying corresponding sequences of S2P6 (I) and 76E1 (J) epitopes of indicated coronaviruses. Red dashed box: S2P6 epitope. The MOW15–22 residue numbering is shown in B and C. (K) Dose-dependent inhibition of NeoCoV pseudovirus entry by antibodies targeting the stem helix (S2P6) or the S2’/fusion peptide (76E1) in HEK293T cells expressing P.pip ACE2. (L) The hACE2-specific antibody h11B11 does not inhibit MERS-CoV pseudovirus entry in Caco2-hACE2–3M cells. n=2 biological replicates for D and E. (M) Dose-dependent inhibition of pcVSV-MOW15–22-S propagation by h11B11. BSA was used as a negative control. Scale bars: 200 [file NIHMS2101199-supplement-Fig_S7.jpg]
