# Supplementary material for: Multiple independent acquisitions of ACE2 usage in MERS-related coronaviruses
Source: Cell. Author manuscript; Available in PMC 2025 Aug 18. (PMC12360793; doi:10.1016/j.cell.2024.12.031)
Supplement: Table S1 [file NIHMS2101199-supplement-Table_S1.pdf]

Table S1, related to Figure 5, Cryo-EM data collection, processing, and model refinement statistics.

	MOW15-22 RBD - P.dav ACE2 PDB 9C6O EMD 45253	MOW15-22 RBD - P.nat.M2 ACE2 PDB 8ZUF EMD 60483	PnNL2018B RBD - P.nat.M2 ACE2 PDB 9DAK EMD 46691
**Data collection and processing**			
Magnification	105,000	50,000	105,000
Voltage (kV)	300	300	300
Electron exposure (e–/Å^2)	60	40	60
Defocus range (μm)	-0.2 to -3.5	-0.5 to -2.5	-0.2 to -3.5
Pixel size (Å)	0.843	0.95	0.843
Symmetry imposed	C1	C1	C1
Initial particle images (no.)	5,091,367	1,844,603	2,063,827
Final particle images (no.)	705,956	244,733	1,555,546
Map resolution (Å)<br>FSC threshold	2.8<br>0.143	3.3<br>0.143	2.4<br>0.143
**Refinement**			
Model resolution (Å)<br>FSC threshold	3.0<br>0.5	3.6<br>0.5	2.5<br>0.5
Map sharpening *B* factor (Å^2)	-101.3	-152.3	-76.2
Model composition<br>Non-hydrogen atoms<br>Protein residues<br>Ligands<br>Water	 6,086<br>777<br>12<br>0	 5,744<br>770<br>18<br>0	 7,303<br>899<br>23<br>144
B factors (Å^2)<br>Protein<br>Ligand<br>Water	 16.53<br>27.55 	 32.31<br>42.05 	 15.85<br>20.98<br>33.10
R.m.s. deviations<br>Bond lengths (Å)<br>Bond angles (°)	 0.010<br>1.049	 0.011<br>1.193	 0.010<br>1.031
**Validation**<br>MolProbity score<br>Clashscore<br>Poor rotamers (%)	 0.98<br>1.47<br>0	 0.94<br>1.8<br>0.24	 0.91<br>1.61<br>0.31
Ramachandran plot<br>Favored (%)<br>Allowed (%)<br>Disallowed (%)	 97.51<br>2.23<br>0.26	 98.55<br>1.19<br>0.26	 98.54<br>1.46<br>0
