# Supplementary material for: Multiple independent acquisitions of ACE2 usage in MERS-related coronaviruses
Source: Cell. Author manuscript; Available in PMC 2025 Aug 18. (PMC12360793; doi:10.1016/j.cell.2024.12.031)
Supplement: Table S2 [file NIHMS2101199-supplement-Table_S2.pdf]

Table S2, related to Figure 5, Receptor-interacting residues in the MOW15-22/P.dav ACE2 and PnNL2018B/P.nat.M2 ACE2 structures.

MOW15-22 residues	PnNL2018B residues
G505	G506
C506	C507
V507	V508
D508	G509
K509	Q510
P511	
Q518	
I519	T515
C520	
I521	L517
P522	
E523	E519
F524	F520
	P521
T526	S522
	F523
D563	D559
S564	S560
T565	V561
D567	D563
I569	
W570	
R571	R567
