# Supplementary material for: Multiple independent acquisitions of ACE2 usage in MERS-related coronaviruses
Source: Cell. Author manuscript; Available in PMC 2025 Aug 18. (PMC12360793; doi:10.1016/j.cell.2024.12.031)
Supplement: Table S3 [file NIHMS2101199-supplement-Table_S3.pdf]

Table S3, related to Figure 5, Virus-interacting residues in the MOW15-22/P.dav ACE2 and PnNL2018B/P.nat.M2 ACE2 structures.

P.dav ACE2	P.nat.M2 ACE2	P.dav ACE2	P.nat.M2 ACE2
P282		Q596	
Y283	F285	S598	
E285	K287		
K286	K288		
P287			
P427			
E428			
D429	D431		
Y430	Y432		
E431	E433		
E433	E435		
I434	I436		
L437	L439		
T534	T536		
	G537		
P536	P538		
H538	H540		
R587	K589		
P588	P590		
N591	S593		
W592	W594		
E595	E597		
